# Comprehensive analysis of a novel RNA modifications-related model in the prognostic characterization, immune landscape and drug therapy of bladder cancer

**DOI:** 10.3389/fgene.2023.1156095

**Published:** 2023-04-12

**Authors:** Ziying Zhang, Peng Chen, Jingping Yun

**Affiliations:** ^1^ State Key Laboratory of Oncology in South China, Collaborative Innovation Center for Cancer Medicine, Sun Yat-sen University Cancer Center, Guangzhou, Guangdong, China; ^2^ Department of Pathology, Sun Yat-sen University Cancer Center, Guangzhou, Guangdong, China; ^3^ Department of Urology, Sun Yat-sen Memorial Hospital, Sun Yat-sen University, Guangzhou, Guangdong, China

**Keywords:** RNA modification, bladder cancer, prognostic model, patient stratification, immunotherapy efficacy

## Abstract

**Background:** Bladder cancer (BCa) is the leading reason for death among genitourinary malignancies. RNA modifications in tumors closely link to the immune microenvironment. Our study aimed to propose a promising model associated with the “writer” enzymes of five primary RNA adenosine modifications (including m^6^A, m^6^A_m_, m^1^A, APA, and A-to-I editing), thus characterizing the clinical outcome, immune landscape and therapeutic efficacy of BCa.

**Methods:** Unsupervised clustering was employed to categorize BCa into different RNA modification patterns based on gene expression profiles of 34 RNA modification “writers”. The RNA modification “writers” score (RMS) signature composed of RNA phenotype-associated differentially expressed genes (DEGs) was established using the least absolute shrinkage and selection operator (LASSO), which was evaluated in meta-GEO (including eight independent GEO datasets) training cohort and the TCGA-BLCA validation cohort. The hub genes in the RMS model were determined *via* weighted gene co-expression network analysis (WGCNA) and were further validated using human specimen. The potential applicability of the RMS model in predicting the therapeutic responsiveness was assessed through the Genomics of Drug Sensitivity in Cancer database and multiple immunotherapy datasets.

**Results:** Two distinct RNA modification patterns were determined among 1,410 BCa samples from a meta-GEO cohort, showing radically varying clinical outcomes and biological characteristics. The RMS model comprising 14 RNA modification phenotype-associated prognostic DEGs positively correlated with the unsatisfactory outcome of BCa patients in meta-GEO training cohort (HR = 3.00, 95% CI = 2.19–4.12) and TCGA-BLCA validation cohort (HR = 1.53, 95% CI = 1.13–2.09). The infiltration of immunosuppressive cells and the activation of EMT, angiogenesis, IL-6/JAK/STAT3 signaling were markedly enriched in RMS-high group. A nomogram exhibited high prognostic prediction accuracy, with a concordance index of 0.785. The therapeutic effect of chemotherapeutic agents and antibody-drug conjugates was significantly different between RMS-low and -high groups. The combination of the RMS model and conventional characteristics (TMB, TNB and PD-L1) achieved an optimal AUC value of 0.828 in differentiating responders from non-responders to immunotherapy.

**Conclusion:** We conferred the first landscape of five forms of RNA modifications in BCa and emphasized the excellent power of an RNA modifications-related model in evaluating BCa prognosis and immune landscape.

## 1 Introduction

BCa is a common malignancy in women and is the fourth most diagnosed malignancy in men globally, with an estimated 500,000 new cases and 200,000 deaths annually (Siegel et al., 2019; Lenis et al., 2020; Richters et al., 2020). Non-muscle-invasive BCa (NMIBC) account for approximately 75% of all newly diagnosed BCa cases. The remaining 25% of BCa cases is muscle-invasive BCa (MIBC) or has already formed metastasis. Transurethral resection of the bladder tumor (TURBT) is the first therapy choice of NMIBC cases, followed by intravesical bacille Calmette-Guerin (BCG) installations or chemotherapy. The 5-year survival rate of NMIBC is about 90%, nevertheless, the postoperative recurrence can occur in over 50% of patients (van Rhijn et al., 2021). MIBC individuals exhibit a poor 5-year survival rate of merely 50% following radical cystectomy and pelvic lymph node dissection, without substantial improvement over the past few decades (van Hoogstraten et al., 2023). The remarkable progresses acquired by immune checkpoint inhibitors (ICIs) (Pembrolizumab and Atezolizumab) have fueled the quest to optimize immunotherapy for both NMIBC and advanced BCa, while only a small percentage of patients display a prominent and durable response to immunotherapy (Lenis et al., 2020). Despite a number of progressions in surgery, radiotherapy, chemotherapy, immunotherapy and targeted therapy that have been achieved in BCa, its prognosis improvement remains a great clinical challenge in BCa. With the development of multi-omic methods, various BCa biomarkers have been revealed (Lu and Zhan, 2018; Miyamoto et al., 2018). Nevertheless, there are no effective and satisfactory biomarkers available for clinical practice to date.

Currently, epigenetic mechanisms implicated in cancer-associated genes and inflammatory genes have gradually been becoming the center of BCa etiology research (Zhang et al., 2019). Epigenetics refers to heritable changes in a cellular phenotype caused by chromosomal alterations that is independent of changes in DNA sequence (Dawson and Kouzarides, 2012). Researches concerning RNA editing, splicing, polyadenylation, and post-transcription are advancing rapidly, thus providing an additional lens through which the essential effects of RNA modifications (also called RNA epigenetics) on modulating BCa development can be unraveled (Sullenger and Nair, 2016; Barbieri and Kouzarides, 2020).

In human cells, RNA modification exists in all nucleotides: A, U, C, and G (Motorin and Helm, 2011). RNA harbors exceeding 170 forms of chemical modifications, such as N^6^-methyladenosine (m^6^A), N^6^,2′-O-dimethyladenosine (m^6^A_m_), N^1^-methyladenosine (m^1^A), N^7^-methylguanosine (m^7^G), and alternative polyadenylation (APA) (Dong and Cui, 2020). As reported, a direct and mutual interplay exists among these modifications. One of the best-characterized examples is that inhibition of m^6^A-catalyzing enzymes leads to global adenosine-to-inosine (A-to-I) editing alterations potentially *via* a disturbance of RNA secondary structure essential for the deamination (Liu et al., 2015; Xiang et al., 2018a). Moreover, a novel molecular axis METTL3/ADAR1/CDK2 conjoining m^6^A and A-to-I that can forcefully alter the scenario of post-transcriptional events and ultimately exerts a pro-oncogenic effect in glioblastoma (Tassinari et al., 2021). Concerning that we are incapable of underlining all types of RNA modifications in our report and adenine is a kind of RNA nucleotide with the most widespread chemical diversities, herein, we primarily concentrated on adenine-associated RNA modifications (m^6^A, m^6^A_m_, m^1^A, APA and A-to-I editing). Above modifications are commonly generated through the activity of enzymes referred to as “writers” (Roundtree et al., 2017).

m^6^A is defined as the methylation occurring at the sixth nitrogen atom of adenine base, which is the most plentiful and better characterized internal RNA modification form in eukaryotic cells (Wei et al., 1975a; Gilbert et al., 2016). This modification is catalyzed *via* m^6^A-methyltransferases complex, including METTL3, METTL14, RBM15, WTAP, VIRMA, ZC3H13, METTL16, CBLL1, and RBM15B (Pendleton et al., 2017; Zaccara et al., 2019). The presence of m^6^A not only influences RNA stability, translational efficiency, and epigenetic function of non-coding RNAs, but also exerts crucial effect on circadian rhythm maintenance and cell cycle modulation, cell differentiation and reprogramming, embryonic stem cell self-renewal, T cell homeostasis, neuronal functions, tumorigenicity and metastasis (Yu et al., 2018; Dong and Cui, 2020).

2′-O-methyladenosine (Am) (as the first nucleotide adjacent to m^7^G cap) is subsequently methylated at the N^6^ position to convert into m^6^A_m_ RNA modification, which is generally mediated by methyltransferase (including PCIF1 and METTL4) (Wei et al., 1975b; Sendinc et al., 2019; Chen et al., 2020a). m^6^A_m_, known as the second most abundant modification in cellular mRNAs and small nuclear RNAs (snRNAs), probably participates in tumor development through modulating RNA splicing, mRNA stability and cap-dependent translation (Dong and Cui, 2020). Specifically, METTL4 as a novel internal m^6^A_m_ methyltransferase for U2 snRNA in human has the capacity to catalyze Am at U2 snRNA position 30 into m^6^A_m_, loss of which broadly impacts various biological pathways, including RNA splicing and cell proliferation (Chen et al., 2020a; Goh et al., 2020; Gu et al., 2020).

m^1^A can be defined as a reversible modification in tRNA, rRNA, mRNA, lncRNA and mitochondrial transcripts, affecting the first nitrogen atom of adenine base (Dominissini et al., 2016; Safra et al., 2017; Scheitl et al., 2020). Multiple m^1^A-methyltransferases as “writers” have been revealed, including TRMT6/61A/61B, TRMT10C, and RRP8 (Zhang and Jia, 2018). The electro-chemical crosstalk caused by positive electrostatic charge of m^1^A can maintain normal function and structure of tRNA. Additionally, m^1^A fosters the translation initiation and tertiary structure of ribosomes while restrains most reverse transcription of RNA, thus modulating the onset and development of diseases (Hauenschild et al., 2015).

APA is a phenomenon that nascent mRNA is cleaved at diverse sites, followed by addition of a poly (A) tail, and further generate multifarious transcript isoforms with diverse lengths of 3′-untranslated region (3′UTR) (Tian et al., 2005; Zhang et al., 2021a). The APA of mRNAs is elicited by multiple subcomplexes, namely, CPSF, CSTF, WDR33, FIP1L1, PCF11, CLP1, and PABPN1 (Schönemann et al., 2014; Tian and Manley, 2017; Brumbaugh et al., 2018; Jang et al., 2019). Because 3′UTR accommodates microRNA (miRNA)-binding sites, APA event is implicated in mRNA stability, translation, and cellular localization. Extensive shortening of 3′UTR has been revealed in a wide variety of tumors, which enables the activation of oncogenes or restrains tumor-suppressor genes in trans through a perturbation of competing endogenous RNA (ceRNA) network, thereby facilitating tumorigenesis (Xia et al., 2014; Xiang et al., 2018b; Park et al., 2018). Disturbance in the expression of APA factors is also detected in diverse malignant tumors, leading to abnormal usage of proximal polyA sites (PAS) (Chu et al., 2019; Fischl et al., 2019).

A-to-I editing is one of the most abundant RNA modification events affecting adenosine in humans, where adenosine deaminase acting on RNA (ADAR) enzymes (including ADAR, ADARB1, and ADARB2) shift adenosine nucleotides towards inosines through the deamination and eventually lead to specific nucleotide alterations at RNA level and changes in the sequence of amino acids in protein without influencing DNA sequence (Xiang et al., 2018a; Eisenberg and Levanon, 2018; Peng et al., 2018). A previous study has reported that A-to-I-edited miR-376a-3p is diminished in glioblastoma, thereby accelerating tumor invasiveness (Choudhury et al., 2012). ADARB1-mediated endogenous and exogenous A-to-I editing in miR-379-5p suppresses tumor proliferation through targeting the apoptosis promoter CD97 (Xu et al., 2019). A-to-I RNA editing in RHOQ is sufficient to confer more aggressive tumor behavior in colorectal cancer (Han et al., 2014). Therefore, A-to-I editing is essential for neoplasia and progressive peculiarity of tumor through modulating site-specific modifications of tumor-associated molecules.

Above five classes of RNA modification “writers” potentially constitute a fundamental and sophisticated regulatory network in BCa, and a thorough comprehending of the network potentially confers a novel insight into the contribution of RNA modification to BCa tumorigenesis. Immune-checkpoint blockade (ICB) is currently on the cutting edge and profiled as the most promising immunotherapeutic strategy in tumor. High tumor mutation burden (TMB) of BCa renders it susceptible to ICB therapy, specifically for monoclonal antibodies targeted programmed cell death-1 (PD-1) and its ligand, PD-L1. Nevertheless, merely lower than 30% of BCa patients yield an objective response from ICB (Balar et al., 2017; Bellmunt et al., 2017). Thus, the ideal approach to screening a cluster of BCa patients who will experience optimal response to the frontline immunotherapy remains to be determined, one of which is to deeply analyze the tumor microenvironment (TME) and mechanism underlying the low response rate to ICB. Compelling and accumulating evidence has demonstrated the crosstalk between immune cells infiltrating in the TME and mRNA modification and associated enzymes. For example, METTL3 deficiency results in the upregulation of IRAKM and subsequently suppresses TLR4 signaling, thus inhibiting macrophage activation (Tong et al., 2021). METTL3 can modulate T cell homeostatic proliferation through targeting IL-7/STAT5/SOCS pathway (Li et al., 2017). METTL3-mediated m^6^A modification also facilitates the translation of CD40, CD80 and cytokine IL-12 transcripts to accelerate dendritic cell (DC) activation (Wang et al., 2019). Thus, RNA modification “writers” are increasingly recognized as an orchestrator to influence homeostasis and function of immune cells in the host. RNA modifications-related score potentially develops into a robust prognostic indicator of immunotherapy.

Precise prognostic model is extremely crucial in tumor immunotherapy. Nevertheless, current studies have principally concentrated on single RNA modification “writer” on account of methodological limitations, while a highly coordinated interaction of various tumor-inhibiting factors is responsible for the antitumor effect of these RNA modification regulators (Dong et al., 2021). Additionally, the potential association between immune landscape of BCa based on the TME and RNA modifications have not been explored in depth and there is no prognostic model based on “writers” of five forms of RNA modifications and their scores in BCa. Thus, a penetrating investigation of dynamic functional network composed of RNA modification regulators and TME components is of extraordinary significance to screen potential subpopulations and exploit preventive, personalized immunotherapy strategies in BCa.

In our study, the transcriptomics data combined with clinicopathological parameters of 1,410 BCa cases were extracted from eight independent Gene Expression Omnibus (GEO) datasets. Firstly, two RNA modification clusters were determined through conducting an unsupervised clustering of gene-expression profiles of 34 RNA modification “writers”. We further correlated RNA modification pattern with the prognosis of BCa and the infiltrating characteristics of multiple immune cells in the TME. Secondly, on the basis of DEGs between two distinct RNA modification patterns, we established RMS model by least absolute shrinkage and selection operator (LASSO) regression, thus individually predicting the prognosis and patients’ responsiveness to chemotherapy drugs, ADCs and ICBs. Then, because the exploration of model-associated molecular mechanism is potentially conducive to its future clinical practice, we investigated the association between the risk score and biological functions, immune characteristics through gene set enrichment analysis (GSEA) and correlation analysis. Additionally, survival analysis, Cox proportional hazards model and receiver operating characteristic (ROC) analysis were performed to evaluate the prognostic performance of RMS model. A nomogram was formulated *via* integrating RMS mdoel and clinicopathological characteristics to predict long-term survival probabilities for BCa patients. The concordance index (C-index), ROC curve, calibration curve analysis, and decision curve analysis (DCA) were applied for assessing the predictive power and accuracy of the nomogram. Ultimately, weighted correlation network analysis (WGCNA) was performed to identify the hub genes associated with RNA modifications. We also validated the hub gene expressions in human BLCA samples compared to tumor-adjacent control samples. In summary, our results emphasize the predictive efficiency of RNA modifications-related model in evaluating the prognosis and therapeutic efficiency of BCa and confer novel insights to elucidate the mechanisms of immune regulation linked to RNA modifications in BCa patients.

## 2 Materials and methods

### 2.1 Data extraction and preprocessing

**FIGURE 1:**
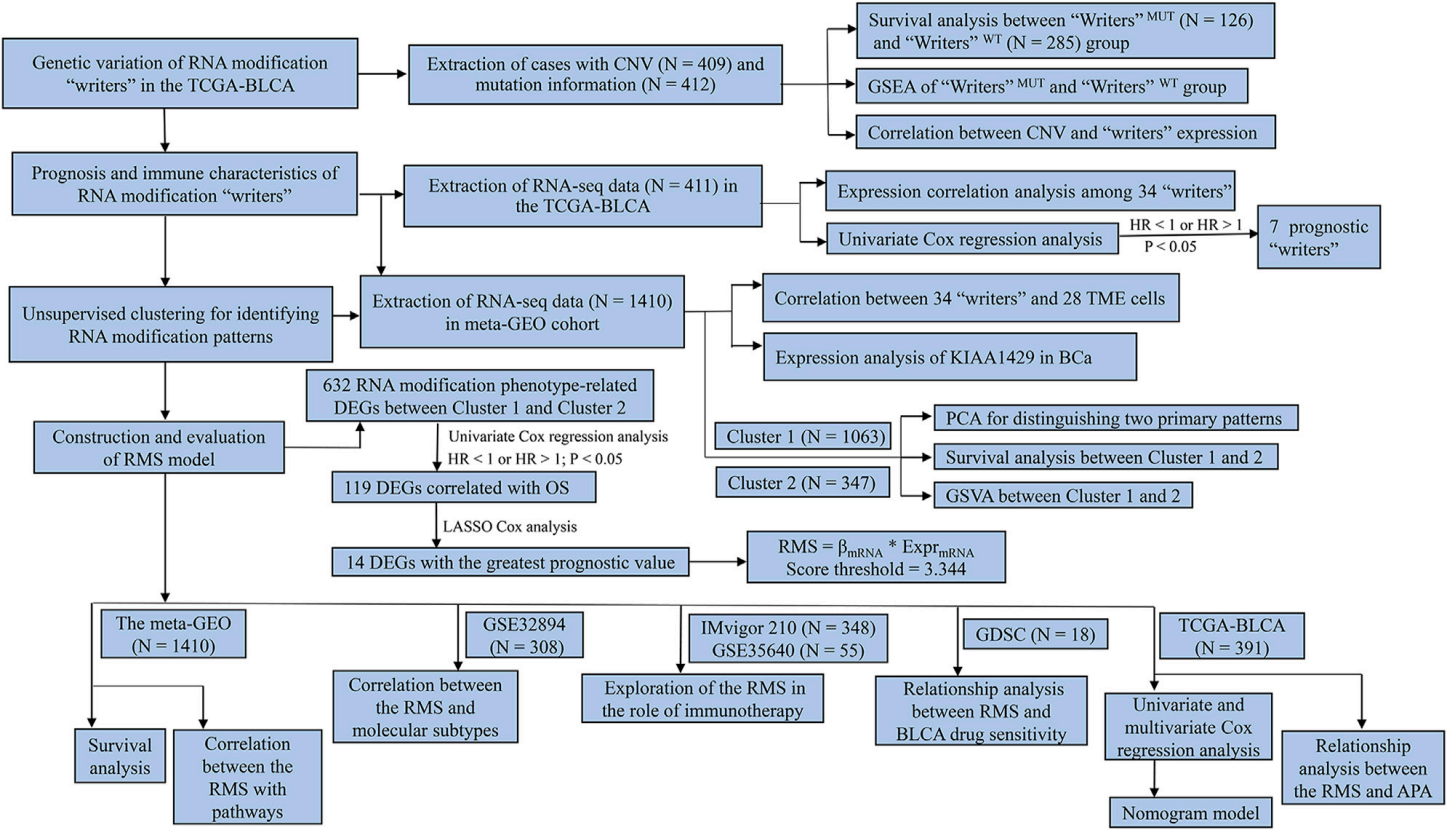
The flow chart of our study.

The program flowchart of our report was illustrated in Figure 1. The public somatic mutation information for 412 BCa samples (workflow type: VarScan2 Variant Aggregation and Masking) was downloaded from The Cancer Genome Atlas-Bladder Urothelial Carcinoma (TCGA-BLCA) (https://portal.gdc.cancer.gov/repository). The somatic copy number variation (CNV) status for 409 BCa cases, and the RNA-sequencing (RNA-seq) data and corresponding clinical datasheets for 411 BCa tissues were extracted from the University of California Santa Cruz (UCSC) Xena browser (https://xenabrowser.net). The clinicopathologic parameters included age, gender, T, N, M classification, tumor stage, histological grade, overall survival (OS) and survival status. RNA-seq data (FPKM values) were further converted into TPM values to make samples more comparable. The waterfall plot that depicted the mutant landscape of TCGA-BLCA cohort was established through “maftools” R package (Mayakonda et al., 2018).

Additionally, the gene expression profiles and detailed clinical annotations of eight BCa-associated Gene Expression Omnibus (GEO) cohorts were extracted from the supplementary files of original manuscript or downloaded from http://www.ncbi.nlm.nih.gov/geo/ through “GEOquery” R package (Davis and Meltzer, 2007), including GSE13507 based on platform GPL6102 (with 188 BLCA samples) (Kim et al., 2010; Lee et al., 2010), GSE32894 based on platform GPL6947 (with 308 BLCA samples) (Sjödahl et al., 2012), GSE32548 based on platform GPL6947 (with 131 BLCA samples) (Lindgren et al., 2012), GSE128959 based on platform GPL6244 (with 200 BLCA samples) (Sjödahl et al., 2020), GSE31684 based on platform GPL570 (with 93 BLCA samples) (Riester et al., 2012; Riester et al., 2014), GSE48075 based on platform GPL6947 (with 142 BLCA samples) (Choi et al., 2014; Guo et al., 2020), GSE104922 based on platform GPL6244 (with 41 BLCA samples) (Therkildsen et al., 2018), GSE83586 based on platform GPL6244 (with 307 BLCA samples) (Sjödahl et al., 2017). The ComBat algorithm of “sva” R Package was utilized for eliminating the batch effects caused by non-biological technical biases (Leek et al., 2012). Eventually, these data from above GEO sets were united into a meta-GEO group (including 1,410 BCa patients) to formulate our RMS model.

### 2.2 Weighted gene co-expression network analysis (WGCNA)

WGCNA can be employed to illuminate the relationship between gene networks modules and clinical phenotype at transcriptome level based on data reduction method and unsupervised classification method (Langfelder and Horvath, 2008). Firstly, the soft threshold power was estimated using nearly scale-free topology to construct a scale-free network. The distance between each gene pair was identified in accordance with the toplogical overlap matrix similarity. Furthermore, hierarchical clustering analysis with the average method and dynamic method was utilized to establish the cluster tree and stratify a variant set of genes into different modules, respectively, respectively. The branches of the cluster tree labeled with a specific color signified one module comprising genes with high correlation. Modules were merged on the condition that their correlation of module eigengene (ME) was over 0.75, implying the similarity of their expression profiles. Pearson’s correlation coefficients and the corresponding *p* values was applied for assessing the correlation between MEs and clinical traits, such as tumor stage, histological grade, and survival status. By convention, one module with the greatest absolute of module significance (MS) was chosen for subsequent analysis. For each module, module membership (MM) was characterized with the correlation coefficient between ME and gene expression profile. Gene Significance (GS) value was applied to quantify the correlation between individual gene and clinical factors (Langfelder and Horvath, 2008). Genes with MM > 0.8 and | GS | > 0.2 were defined as hub genes in the module. In our study, the “WGCNA” R package was performed to establish a co-expression network of 5,657 prognosis-associated genes in 411 BCa patients with clinicopathological parameters (Zhang et al., 2021b).

### 2.3 Immunohistochemistry (IHC)

Samples were collected from BCa cases who conformed to the following criteria (Richters et al., 2020). Patients were clinically and pathologically diagnosed with BCa (Siegel et al., 2019). None of cases was performed by radiotherapy or chemotherapy before surgery (Lenis et al., 2020). Paired adjacent non-neoplastic bladder tissues were available for contrast. All tissues were acquired from Sun Yat-sen University Cancer Center and immediately fixed with formalin. The samples were embedded in paraffin for the construction of tissue microarray (TMA) that included 84 paired BCa samples. Prior to participating in this study, all patients received written informed consent. The project was approved by the Ethics Committee of Sun Yat-sen University Cancer Center.

The BCa TMA was dewaxed with xylene and further blocked endogenous peroxidase activity in 3% hydrogen peroxide solution. Antigen retrieval was conducted through boiling the samples in sodium-citrate buffer (pH 6.0) for 3 min. The TMA was incubated overnight with primary antibodies (anti-KIAA1429: 1:500, # 25712-1-AP, Proteintech, United States) at 4°C overnight. After incubating with secondary antibody at room temperature for 30 min, the TMA was counter-stained with hematoxylin, dehydrated and covered. The degree of immunostaining of the TMA was evaluated by two independent pathologists blinded to the histopathological characteristics of the samples. The proportion of positively stained cells was scored on a scale of 0–4 (0%, 1%–25%, 26%–50%, 51%–75%, and 76%–100%). The staining intensity was scored with four scoring levels: 0 (negative), 1 (weak), 2 (medium) and 3 (strong). The staining score was staining by multiplying the proportion of positively stained cells with the intensity score. The total scores were relatively stratified into three grades, <3 scores, 3 to 6 scores, and >6 scores, which corresponded to negative, weak positive and strong positive staining, respectively.

### 2.4 Real-time quantitative PCR (RT-qPCR)

Based on the manufacturer’s instructions, TRIzol reagent (Invitrogen, Carlsbad, CA) was utilized to isolate total RNA from tissues. Then, cDNA was generated by reverse transcription using the HiScript II Q RT SuperMix Kit (Vazyme Biotech, Nanjing, China). RT-qPCR was performed using the SYBR Green approach (Roche, Basel, Switzerland) in a Roche LightCycler 480 II PCR system (Roche Diagnostics, Rotkreuz, Switzerland). GAPDH was applied for normalizing target gene expression. The RT-qPCR primer sequences were: KIAA1429, forward 5′- TCG​ATA​GGT​TGG​GAA​GCC​TGG-3′ and reverse 5′- TAC​CAG​CCT​CTT​AGC​ACC​AGA-3′.

### 2.5 Western blot assay

RIPA Lysis Buffer (Beyotime, Shanghai, China) was used to extract total protein from fresh tissue samples and the BCA Protein Assay Kit (Thermo Fisher Scientific, Waltham, United States) was applied for measuring protein concentrations. Subsequently, total protein was separated *via* an SDS-polyacrylamide gel and was further moved to a polyvinylidene fluoride membrane. The membrane was incubated overnight at 4°C with primary antibody rabbit monoclonal anti-KIAA1429 (# 25712-1-AP, Proteintech, United States; 1:1,000 dilution) and mouse monoclonal anti-beta-tubulin (# sc- 5274, Santa Cruz, United States; 1:1,000 dilution).

### 2.6 Collection of clinical datasets with immunotherapy

Three immunotherapeutic cohorts with accessible genomic/transcriptomic data and sufficient clinical annotations were enrolled into our report to investigate the association between the RMS and efficacy of immunotherapy (Richters et al., 2020). IMvigor210 cohort, advanced urothelial carcinoma (UC) with atezolizumab (anti-PD-L1 antibody) treatment (Mariathasan et al., 2018; Siegel et al., 2019) Snyder UC cohort, patients with locally advanced or metastatic UC (mUC) treated with atezolizumab (Snyder et al., 2017; Lenis et al., 2020) Montoya melanoma cohort, advanced melanoma patients underwent MAGE-3 antigen-based immunotherapy (Ulloa-Montoya et al., 2013).

For IMvigor210 cohort, according to the Creative Commons 3.0 License, the gene expression data and detailed clinical annotation were downloaded from http://research-pub.gene.com/IMvigor210CoreBiologies. The raw data were normalized through “edgeR” R package and were subsequently converted to TPM values. Similarly, data of Snyder UC cohort were extracted from http://doi.org/10.5281/zenodo.546110. Furthermore, RNA-seq and clinical information from Montoya melanoma cohort were deposited in GSE35640 (N = 55).

### 2.7 Unsupervised clustering analysis of RNA modification “writers”

To identify the optimal number of clusters, we utilized unsupervised consensus hierarchical clustering algorithm through “ConsensuClusterPlus” R package, to perform clustering analysis of 34 RNA modification “writers” of 1,410 BCa samples in the meta-GEO cohort (Wilkerson and Hayes, 2010). The robustness of above stratification was identified *via* the consensus clustering algorithm with 1,000 times repetitions.

### 2.8 Gene set variation analysis (GSVA) and gene ontology (GO) analysis

GSVA was conducted using “GSVA” R package, thus depicting the differences in the enrichment of signaling pathways between diverse RNA modification patterns (Hänzelmann et al., 2013). The well-acknowledged biological signatures were acquired from the Hallmarker gene set [curated from the Molecular Signature Database (MSigDB) v7.1] (Subramanian et al., 2005) and Mariathasan *et al.* established gene set (download from http://research-pub.gene.com/IMvigor210CoreBiologies) (Mariathasan et al., 2018). GO functional annotation for 34 RNA modification enzyme genes were identified through “clusterProfiler” R package with a threshold of false discovery rate (FDR) < 0.05 (Yu et al., 2012; Zhang et al., 2021b).

### 2.9 Identification of RNA modification phenotype-related DEGs

A total of 1,410 BCa patients were stratified into two different RNA modification patterns in line with the preceding consensus clustering algorithm. RNA phenotype-related DEGs between Cluster 1 and Cluster 2 were determined using the empirical Bayesian method of “limma” R package (Ritchie et al., 2015). DEG with│log_2_fold change (FC)│> 1 and an adjusted *p*-value <0.001 was considered as the significance criteria.

### 2.10 Construction and validation of the RMS model

We further established a scoring model to assess RNA modification pattern of each BCa patient—the signature of RNA modification “writers”, and we termed as the RMS. Initially, univariate Cox regression analysis was carried out to estimate the HR of RNA phenotype-related DEGs using “survminer” R package. Among the resulting DEGs with significantly prognostic power (*p* < 0.05) based on univariate Cox regression analysis, pivotal prognostic DEGs were further identified by the LASSO with L1-penalty using “glmnet” R package (Engebretsen and Bohlin, 2019), ultimately formulating the RMS model. The LASSO method determines interpretable prediction rules that can resolve the collinearity and overfitting problem, which is applied to build models when there are plenty of correlated covariates (Gui and Li, 2005). In this algorithm, a sub-selection of RNA phenotype-related DEGs associated with BCa patients’ prognosis was identified through shrinkage of the regression coefficient and fewer parameters with a weight of non-zero ultimately remained. Thus, LASSO Cox regression reinforced the prediction accuracy of the model through diminishing the number of DEGs (Long et al., 2019). Subsequently, RNA phenotype-associated prognostic model was established through multiplying the regression coefficient derived from LASSO Cox regression by the expression level of each DEG. We defined the RMS of each case in the meta-GEO using the following formula: RMS = β_mRNA1_ * Expr_mRNA1_ + β_mRNA2_ * Expr_mRNA2_ + ⋯⋯ + β_mRNAn_ * Expr_mRNAn_, where Expr was the expression level of DEG and β was the Cox regression coefficient. Eventually, we categorized all BCa cases in the meta-GEO dataset into RMS-high and -low groups using the median risk score. To reap a uniform cutoff value to classify the cases into high and low RMS groups, a normalization for the expression values of DEGs were normalized with standard deviation (SD) = 1 and average value = 0 in the TCGA-BLCA and meta-GEO cohort. To further validate the RMS model, the risk score calculation for each patient and the stratification of patients in the TCGA-BLCA was determined according to the same formula and the identical cutoff value derived from the meta-GEO cohort, respectively.

### 2.11 Construction and validation of nomogram model

All statistically significant clinicopathological characteristics identified by multivariate Cox analysis were included to build the prognostic nomogram model with “rms” R package, thereby estimating survival probability of BCa individuals (Chen et al., 2020b; Zhang et al., 2021b). The concordance index (C-index) and calibration curves were applied for assessing the prediction accuracy of the nomogram. The closer to 1 the C‐index is, the more accurate the predictive efficiency of nomogram is (Zhang et al., 2021b). The time-dependent receiver operating characteristic (ROC) curve with an area under the curve (AUC) value was formulated by “survivalROC” R package, thereby evaluating the predictive performance of the nomogram (Chen et al., 2020b).

### 2.12 Analysis of RMS-related DEGs in pan-cancer

The online GSCALite website (http://bioinfo.life.hust.edu.cn/web/GSCALite/) was applied for exploring the mRNA expression, CNV, and pathway activity of RMS-related DEGs in pan-cancer (Liu et al., 2018).

### 2.13 Characterization and survival analysis of APA events between RMS-high and -low group

APA profile in BCa was downloaded from Synapse (https://www.synapse.org/, syn7888354) (Xiang et al., 2018b). The DaPars algorithm (https://github.com/ZhengXia/DaPars) has been used to estimate the relative polyA site usage in 3′UTR caused by APA through the Percentage of Distal polyA site Usage Index (PDUI) that is a quantitative index to determine 3′UTR lengthening (positive index) or shortening (negative index) (Xia et al., 2014). The Wilcoxon rank-sum test was used to compare the differences in 3′UTR between RMS-high and -low group, and we considered *p* < 0.05 and |ΔPDUI| = |PDUI _RMS-high_ − PDUI _RMS-low_ | > 0.1 as statistically significant.

Univariate Cox regression analysis was performed to determine the prognostic significance of each differential APA event between RMS-high and -low group using “survival” R package. All BCa patients in the TCGA were stratified into two groups in accordance with PDUI value, and Kaplan-Meier curve with log-rank test was established to assess their survival difference.

### 2.14 Correlation analysis of the RMS and drug sensitivity

We acquired RNA-seq data of 18 kinds of BCa cell lines, AUC values as drug response measurements of antineoplastic drugs in BCa cell lines, and targets or pathways of drugs from Genomics of Drug Sensitivity in Cancer (GDSC, https://www.cancerrxgene.org/) (Yang et al., 2013). Spearman correlation analysis was conducted to estimate the association between drug sensitivity and the RMS, with the cutoff values of | Spearman Correlation Rs | > 0.2 and FDR <0.05.

### 2.15 Statistical analysis

Difference analysis was performed by Wilcoxon rank-sum test. Survival curve was established using Kaplan-Meier method, and log-rank test was utilized to estimate the significance of differences. Tumor and Immune System Interaction Database (TISIDB, http://cis.hku.hk/TISIDB/; up to 15 March 2021) was utilized to unravel the correlation between the abundance of various tumor-infiltrating immune cells and the expression of RNA modification “writers” genes (Ru et al., 2019). The “pROC” R package was utilized to formulate ROC curve with corresponding AUC value, thus verifying the predictive power of the RMS model. Univariate Cox analysis was applied for calculating the HR value of RNA phenotype-associated DEGs. All significant independent prognostic factors were identified *via* multivariable Cox regression analysis using “survminer” R package. The Benjamini–Hochberg algorithm was used to convert *p*-value to FDR (Glickman et al., 2014). All statistical analysis was conducted using R 3.6.2 software, and *p* < 0.05 were considered statistically significant.

## 3 Results

### 3.1 Genetic and transcriptional landscape of five types of RNA modification “writers” in BCa

In accordance with published articles depicting RNA modification (Elkon et al., 2013; Zaccara et al., 2019; Dong and Cui, 2020; Marceca et al., 2021), a catalog of 34 RNA modification “writers” were enrolled into our study, including 9 m^6^A modification “writers” (METTL3, METTL14, RBM15, WTAP, KIAA1429, ZC3H13, METTL16, CBLL1 and RBM15B), 2 m^6^A_m_ modification “writers” (PCIF1 and METTL4), 5 m^1^A modification “writers” (TRMT6, TRMT61A, TRMT10C, TRMT61B, RRP8), 15 APA modification enzymes (CPSF1/2/3/4, NUDT21, CPSF6/7, CSTF1/2/3, WDR33, FIP1L1, CLP1, PCF11, PABPN1), and 3 A-to-I modification enzymes (ADAR, ADARB1 and ADARB2) (Supplementary Table S1).

**FIGURE 2:**
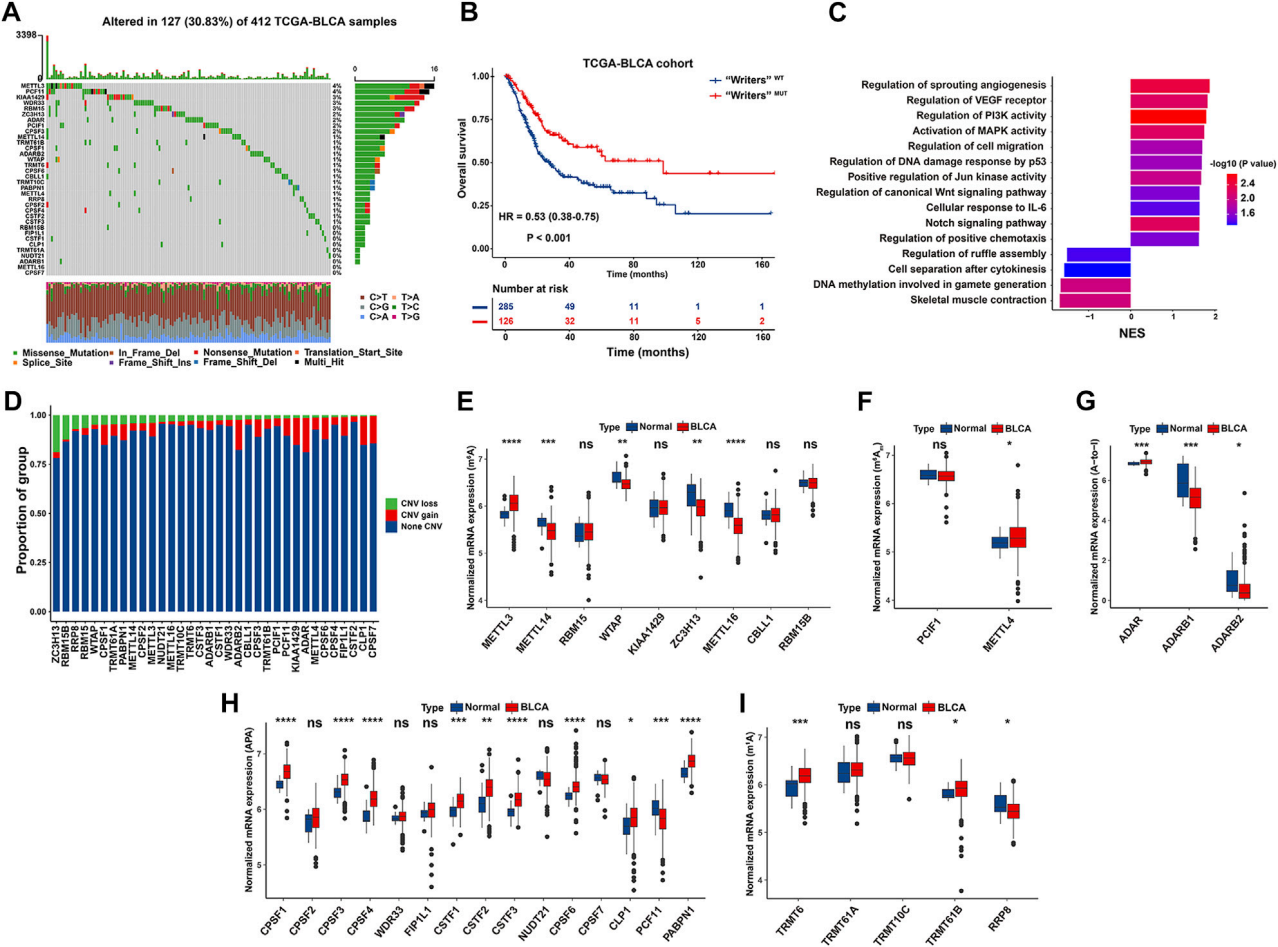
Genetic and transcriptional characteristics of RNA modification “writers” in the TCGA-BLCA cohort. **(A)** Waterfall plot showing mutation frequency of 34 “writers” in 412 BCa cases. The upper and the right bar chart represented the TMB for individual sample, and the proportion of mutation type of each “writer”, respectively. **(B)** Kaplan-Meier curve of OS in “Writers” ^MUT^ and “Writers” ^WT^ BCa patients. **(C)** Bar chart depicting significantly biological pathways enriched in “Writers” ^WT^ (Right) or “Writers” ^MUT^ (Left) BCa patients by GSEA. **(D)** Bar chart depicting CNV frequency of 34 RNA modification “writers”. **(E–I)** Boxplot representing mRNA levels of 34 “writers” between normal tissues and BCa samples, with *p* values derived from Wilcoxon rank sum test.

To delineate genetic landscape of RNA modification “writers” in BLCA, we evaluated the frequency of non-silent somatic mutations in 34 “writers” based on mutational information of the TCGA-BLCA database. Specifically, 127 of 412 BLCA cases (30.83%) experienced mutations of RNA modification “writers”. METTL3 displayed the greatest mutation frequency (4%), followed by PCF11 (4%), KIAA1429 (3%), and WDR33 (3%). While the mutation frequency of ADARB1, METTL16 and CPSF7 was extremely low in BCa samples. Missense mutation constitutes the predominant type of mutations for each writer (Figure 2A).

We demonstrated that BCa patients with mutant “writers” exhibited a significantly prolonged OS than those without mutation (HR = 0.53, 95% CI: 0.38–0.75, *p* < 0.001) (Figure 2B), indicating that genetic alteration of “writers” potentially exerts a functional effect towards BCa tumorigenicity. GSEA was carried out to decipher biologic themes specific for “writers” mutated (“Writers” ^MUT^) group (N = 126) and “writers” wild‐type (“Writers” ^WT^) group (N = 285) of patients in the TCGA-BLCA. “Writers” ^WT^ group was markedly enriched in carcinogenic activation pathways, such as angiogenesis, PI3K signaling, MAPK activity, P53 signaling, Jun kinase activity, and canonical Wnt signaling pathway (Figure 2C; Supplementary Tables S2, S3). Hence, the mutation of “writers” is prone to trigger functional alterations with prognostic significance in BCa.

We also investigated CNV alteration frequency of these “writers” and unraveled that ADAR, ADARB2, CLP1, and CPSF7 had a relatively high frequency of CNV amplification, while ZC3H13, RBM15B and RRP8 experienced a widespread frequency of CNV deletion (Figure 2D). To determine whether CNV plays a considerable role in the expression of RNA modification “writers” in BCa patients, we attempted to assess the mRNA level of “writers” between normal and BCa tissues in the TCGA database. As depicted in Figures 2E–I, a large proportion of enzyme-associated genes displayed relatively greater mRNA expression in BCa than normal tissues, highlighting the profound function of these “writers” in the occurrence and development of BCa. Moreover, RNA modification “writers” with CNV gain (such as ADAR, CLP1, and CPSF6) were significantly upregulated in BCa samples than normal tissues. On the contrary, the expression of “writers” genes with CNV loss (including ZC3H13 and RRP8) was significantly diminished in BCa *versus* normal bladder tissues. Notably, certain “writers” (such as ADARB2 and PCF11) with widespread frequency of CNV gain harbored decreased mRNA level in BCa compared to adjacent non-tumor tissues (Figures 2D, G, H).

To further elucidate the association between CNV values and mRNA expression in BCa samples, we stratified the TCGA-BLCA cohort into three groups according to CNV values of four “writers” characterized with exceeding 5% of CNV loss in BCa tissues, including CNV gain, CNV loss and non-significant alteration of CNV. Concretely, ZC3H13, RBM15B, RRP8, and RBM15 with CNV gain exhibited dramatically enhanced mRNA level than that with CNV loss, respectively. Nevertheless, the mRNA levels of above “writers” were significantly decreased or without remarkable alteration in CNV loss group compared with those in non-tumor samples (Supplementary Figure S1A). Thus, CNV alteration partially explains why there is differential mRNA expression between tumor and normal samples (Sebestyén et al., 2016). Additional parameters, such as DNA methylation and transcription factors, are also endowed with the robust capacity to orchestrate gene expression in tumorigenesis (Lambert et al., 2018; da Rocha and Gendrel, 2019).

### 3.2 Prognosis and immune characteristics of RNA modification “writers” in BCa

**FIGURE 3:**
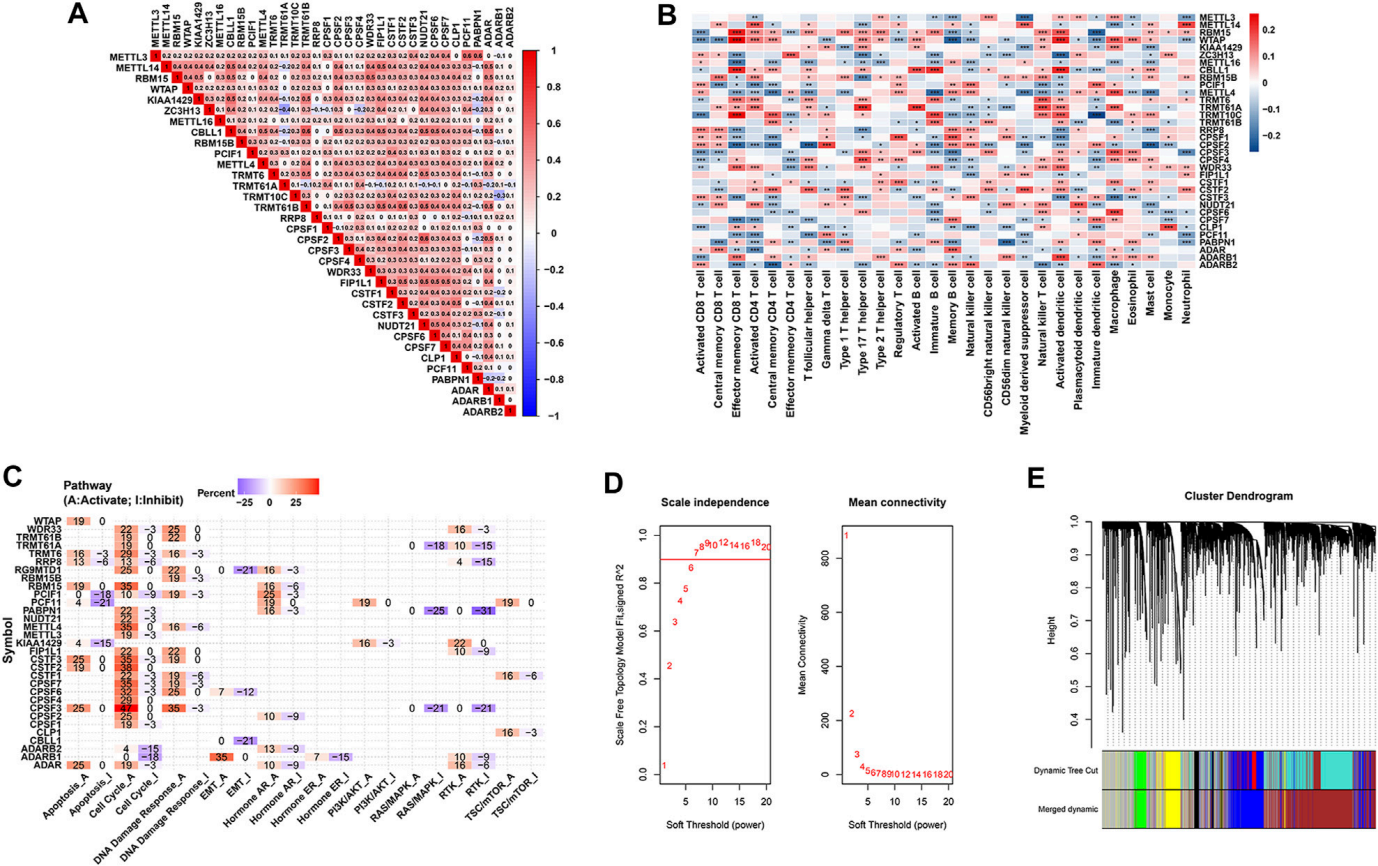
Prognosis and immune characteristics of RNA modification “writers” in BCa samples. **(A)** Heatmap showing the positive and negative correlations among 34 RNA modification “writers” in the TCGA-BLCA cohort. **(B)** Heatmap displaying the positive and negative correlations between 34 RNA modification “writers” and infiltrating proportions of 28 immune cells in the meta-GEO cohort. **(C)** Heatmap displaying “writers” with inhibitory (Blue) or activated (Red) functions in multiple pathways in BCa. **(D)** The scale-independence index and the mean connectivity for diverse soft threshold powers of the WGCNA. **(E)** Cluster dendrogram of prognosis-associated genes in the TCGA-BLCA using WGCNA method. Each branch in the figure and each color below represented one gene and one co-expression module, respectively.

Pairwise correlation analysis demonstrated that not only RNA modification “writers” in the same functional category exhibited a significant correlation in expression, but also a significant correlation was presented among mRNA levels of different category of “writers”. For example, BCa samples with high expression of A-to-I “writer” gene ADAR were accompanied by increased mRNA levels of eight m^6^A “writer” gene, including METTL14, RBM15, WTAP, VIRMA, ZC3H13, METTL16, CBLL1 and RBM15B, indicating a potential crosstalk between m^6^A and A-to-I modification in BCa (Figure 3A). Whether co-expression phenomenon of these “writer” genes hints a functional correlation is a topic that motivates us to pursue further investigation. Additionally, prognosis analysis demonstrated that seven of 34 RNA modification “writers” were prognostic parameters of BCa cases in the TCGA-BLCA. BCa patients with higher KIAA1429 expression had a shorter survival time (HR = 1.35, 95% CI: 1.01–1.82, *p* = 0.0447) (Supplementary Figure S1B).

To further comprehensively expound the expression pattern of 34 “writers” in BCa, 1,410 BCa patients from eight independent GSE sets were combined into a meta-GEO group in our study (Supplementary Table S4). As revealed in Figure 3B, BCa patients with high KIAA1429 expression were characterized with an increased proportion of macrophage and Type 17 T helper cells (Th17 cells). We also mined the GSCALite web server and found that KIAA1429 expression was negatively related to apoptosis, and positively associated with PI3K/Akt and RTK pathways (Figure 3C).

### 3.3 WGCNA used for the screening of KIAA1429

**FIGURE 4:**
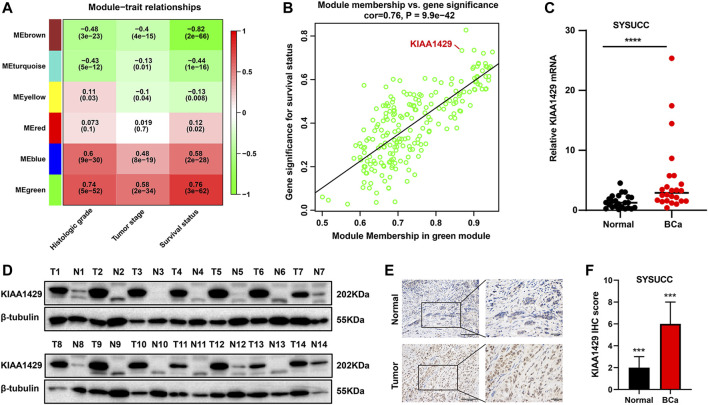
Prognosis and expression characteristics of KIAA1429 in BCa samples. **(A)** Heatmap illustrating the modules‐trait correlation between module eigengenes and clinical traits using WGCNA method. **(B)** Scatter plots showing the correlation between GS and MM of genes in green module using WGCNA method. **(C)** qRT-PCR analysis revealing mRNA expression levels of KIAA1429 in SYSUCC samples. GAPDH was served as an internal control. **(D)** Western blot analysis showing increased KIAA1429 protein levels in BCa tissues, compared to the paired adjacnt normal bladder urothelial tissues. **(E)** Representative IHC images exhibiting KIAA1429 expression in BCa samples and adjacent normal bladder urothelial tissues. Original magnification, ×200. **(F)** IHC score of KIAA1429 staining in BCa tissues and corresponding non-cancerous samples.

Considering that the broad impacts of m^6^A “writer” KIAA1429 on epigenetic modifications, we further emphatically discussed the prognostic significance of KIAA1429 in BCa. Initially, a total of 5,657 prognosis-associated genes were extracted in 411 BCa patients, among which 3,497 genes were associated with favorable prognosis (HR < 1, *p* < 0.05) and 2,160 genes were related to unfavorable prognosis (HR > 1, *p* < 0.05). To select pivotal hub genes associated with BCa progression, above 5,657 prognosis-associated genes were applied to cluster analysis by the “WGCNA” R package. On the basis of the standard scale‐free network distribution, we carefully set the soft threshold power value as 7 to formulate a hierarchical clustering tree (dendrogram) of 5,657 genes (Figure 3D). According to the dynamic tree cut algorithm, the least gene number of each module and the minimum cut height was 50 and 0.25, respectively. The correlation of characteristic genes in integrated modules was over 0.9. We identified six co-expression modules containing all genes based on their degree of connectivity. The gray section represented background genes that did not belong to any modules (Figure 3E). We ultimately assessed the correlation between MEs and clinical traits, including TNM stage, histological grade and survival status. Specifically, the green module was characterized with the strongest positive correlation with survival status (r = 0.76, *p* < 0.0001), which was considered as the most significant module to the prognosis of BCa (Figure 4A). There were three RNA modification “writers” in the green module, of which MM value and the absolute value of GS of KIAA1429 ranked first (*p* < 0.0001) (Figure 4B). Thus, KIAA1429 can be defined as one hub gene significantly related to survival status and BCa prognosis in the green module.

### 3.4 KIAA1429 expression in bladder cancer

To clarify the role of KIAA1429 in BCa, we used qRT-PCR to examine the expression of KIAA1429 in 20 pairs of human primary BCa tissues and paracancerous normal samples. Upregulation of KIAA1429 mRNA was revealed in BCa tumor samples compared with the corresponding non-cancerous samples (Figure 4C). Consistent with the findings of qRT-PCR assay, KIAA1429 at the protein levels was overexpressed in BCa through Western blotting test (Figure 4D). Additionally, the KIAA1429 protein was detected using IHC in 84 paired BCa clinical tissues that underwent radical cystectomy, and its expression was significantly elevated in BCa tissues (Figures 4E, F).

### 3.5 Immune landscape of RNA modification-associated patterns in BCa

**FIGURE 5:**
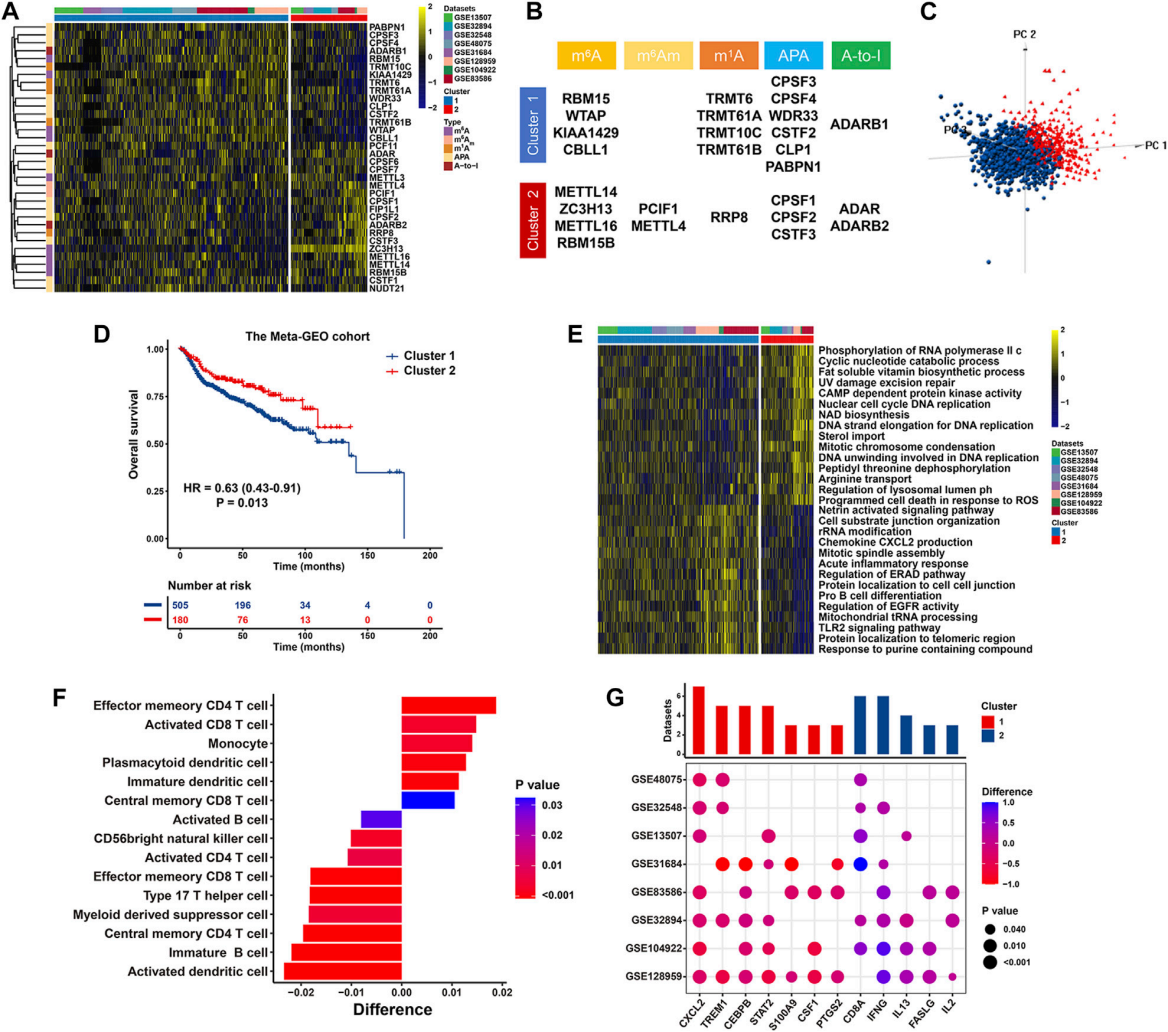
RNA modification patterns and their biological significance in the meta-GEO cohort. **(A)** Heatmap presenting unsupervised clustering results of 34 RNA modification “writers” in eight independent BCa cohorts. Each column and row represented patients and RNA modification “writers”, respectively. **(B)** Specific distribution of 34 RNA modification “writers” enriched in two primary patterns. **(C)** PCA illustrating the expression of 34 RNA modification “writers” to distinguish two primary patterns in 1,410 BCa patients. **(D)** Kaplan-Meier curve of patients’ OS in two RNA modification patterns. **(E)** Heatmap displaying the difference in relatively activated biological processes between two distinct RNA modification patterns by GSVA. **(F)** Bar chart showing the proportion of immune cells between two clusters. Difference >0 or <0 represented the immune cells enriched in Cluster 2 or Cluster 1, respectively. **(G)** Difference in the expression of MDSCs and activated CD8^+^ T cells marker genes between two clusters. The upper bar chart represented the number of datasets that were significantly different between Cluster 1 and Cluster 2. The color and size bubble illustrated the difference in each GEO, and the statistical significance of difference, respectively. Difference >0 or <0 implied greater expression of immune cell marker genes in Cluster 2 or Cluster 1, respectively.

On the basis of the expression profiles of 34 RNA modification “writers” in the meta-GEO cohort, we conducted unsupervised consensus clustering to stratify BCa patients with qualitatively varying RNA modification patterns into two distinct clusters, eventually including 1,063 cases in Cluster 1 and 347 cases in Cluster 2, respectively (Figure 5A and Supplementary Figure S2A). Specifically, Cluster 1 had significantly increased presence of RBM15, WTAP, KIAA1429, TRMT6, TRMT61B, CPSF3, CPSF4, WDR33 and ADARB1, while Cluster 2 was characterized with elevated level of METTL14, ZC3H13, METTL16, PCIF1, METTL4, RRP8, CPSF1 and ADARB2 (Figure 5B and Supplementary Figure S2B). Furthermore, based on above-identified clusters, BCa patients in Cluster 1 and Cluster 2 were visibly separated into two discrete groups using three dimensional PCA, emphasizing that BCa cases are well stratified in line with the mRNA levels of 34 RNA modification “writers” (Figure 5C). Survival analysis for two primary RNA modification subtypes demonstrated that compared with Cluster 1 modification pattern, Cluster 2 pattern was linked to significantly prolonged survival (HR = 0.63, 95% CI = 0.43–0.91, *p* = 0.013) (Figure 5D). We further conducted GSVA to investigate the biological behaviors of above different RNA modification patterns. The carcinogenic activation pathways, including the epidermal growth factor activated receptor activity, TLR-2 signaling pathway, chemokine CXCL2 production and cell adhesion, were enriched relative to Cluster 1, indicating an inflammation activation and tumorigenesis status in Cluster 1. While Cluster 2 represented a metabolic or biosynthetic activation phenotype, prominently enriched in pathways related to the cyclic nucleotide catabolic process, CAMP-dependent protein kinase activity and DNA replication (Figure 5E and Supplementary Table S5).

We also unraveled the discrepancies concerning the compositions of tumor‐infiltrating immune cells between two RNA modification clusters. Significant difference in immune cell fractions in two primary patterns were summarized in Supplementary Tables S6, S7. As revealed in Figure 5F and Supplementary Figure S2C, Cluster 1 was characterized with an increased proportion of MDSCs with formidable immunosuppressive property (*p* < 0.0001) and Th17 cells (*p* < 0.0001). Conversely, cases in Cluster 2 exhibited prominent infiltration of activated CD8^+^ T cells with pronounced antitumor activity (*p* < 0.001), effector memory CD4^+^ T cells (*p* < 0.0001), and central memory CD8^+^ T cells (*p* = 0.032). Consistently, compared with cases in Cluster 2, those in Cluster 1 had significantly increased levels of MDSC marker genes (including STAT2, S100A9, CXCL2, CSF1, PTGS2, TREM1, CEBPB) while significant downregulation of activated CD8^+^ T cell marker genes (such as CD8A, IFN-γ, IL-13, and FASLG) (Figure 5G). In summary, RNA modification patterns exert an effect on the proportions of infiltration by specific immune cell types while fail to change the types of infiltrating immune cells.

### 3.6 Establishment of the RMS model and its clinical significance in BCa

We further determined 632 RNA modification phenotype-associated DEGs between Cluster 1 and Cluster 2. The biological processes with significant enrichment associated with these DEGs were enriched in purine nucleotide metabolic process, methylation, DNA replication, and cell cycle G1/S phase transition, all of which were closely related to RNA processing (Supplementary Table S8 and Supplementary Figure S3A). In consideration of the heterogeneity and complexity of RNA modification, we attempted to establish a risk score system named the RMS (RNA Modification “Writers” Score). Firstly, we confirmed that 119 of 632 DEGs was significantly correlated with OS through univariate Cox regression analysis (Supplementary Table S9). To reveal potential DEGs with the optimal prognostic performance, we utilized LASSO Cox analysis, and 14 DEGs were incorporated into our subsequent analysis (Supplementary Table S10; Supplementary Figures S3B, C). Furthermore, we performed normalization of the expression levels of 14 DEGs in the TCGA and meta-GEO cohort with average value = 0 and SD = 1, thus acquiring a uniform cutoff value as stratified standard. Then, we quantified the RNA modification status of each BCa patient by weighting the normalized mRNA level of each DEG to the regression coefficient. The concrete formula was the following: RMS = 0.2062 * Expr _IFNLR1_ + 0.1822 *Expr_PCDHB11_ + (−0.1428) * Expr _TIMM21_ +……+ 0.0057 * Expr _CRELD1_+0.0028* Expr _FOXG1_. Ultimately, we calculated the RMS for each BCa case in the meta-GEO and stratified all cases into RMS-high and -low cohorts based on the median value (3.344) (Supplementary Table S11).

**FIGURE 6:**
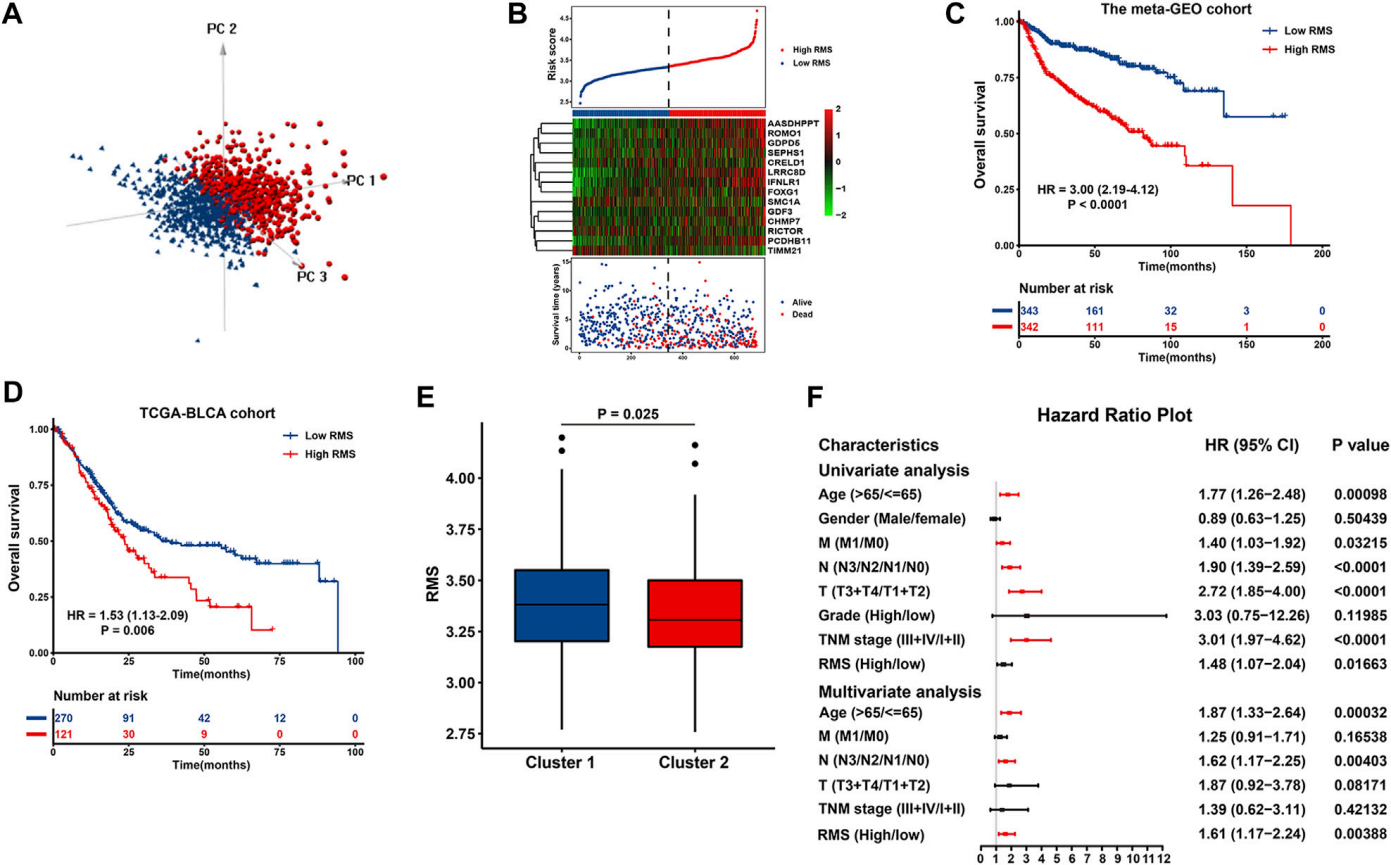
Construction and evaluation of the RMS model in BCa. **(A)** PCA exhibiting a remarkable distinction between cases with high or low risk score. **(B)** The risk score distribution, heatmap showing the expression of 14 DEGs, and survival status for each BCa case in the meta-GEO group. The black dotted line indicated the RMS cutoff to classify cases into low‐ and high‐risk groups. Kaplan-Meier curve revealing OS difference between RMS-high or -low patients in **(C)** the TCGA and **(D)** meta‐GEO group. **(E)** Boxplots describing the RMS of two RNA modification patterns in the meta-GEO cohort. **(F)** Forest plot of Cox regression analysis to evaluate the relationship between the RMS and clinicopathological parameters.

As showed in Figure 6A, the RMS classified BCa cases with high or low risk score into two discrete sections, highlighting that the RMS distribution of BCa cases in the low‐risk group was greatly different from those with high risk score. There was a high degree of consistency among the risk score distribution, the heatmap of 14 prognostic DEGs’ expression and survival status of BCa case in the meta-GEO cohort (Figure 6B). Notably, the cutoff point (3.344) also served as a classification indicator in the TCGA-BLCA cohort. Kaplan-Meier curve revealed that high RMS was significantly correlated with more unfavorable clinical outcome of BCa cases in the meta-GEO (HR = 3.00, 95% CI: 2.19–4.12, *p* = 1.06e^−11^) (Figure 6C) and TCGA-BLCA cohort (HR = 1.53, 95% CI: 1.13–2.09, *p* = 0.006) (Figure 6D). Additionally, patients with high RMS had a shorter disease-specific survival (DSS) than their RMS-low counterparts in GSE32894 (HR = 18.3, 95% CI: 4.30–77.6, *p* < 0.001) (Supplementary Figure S3D). In GSE31684 dataset with recurrence data, the RMS was significantly negatively associated with recurrence-free survival (RFS) (HR = 2.02, 95% CI: 1.06–3.88, *p* = 0.033) (Supplementary Figure S3E). We also investigated the correlation between the RMS and cluster classifier to evaluate the RMS model’s accuracy. As revealed in Figure 6E, the RMS of BCa samples in Cluster 1 was significantly higher than that of cases in Cluster 2 (*p* = 0.025). We found that 579 out of 742 (78.03%) samples with high RMS were overlapped with the samples in Cluster 1, and 184 out of 668 (27.54%) cases in RMS-low group overlapped with the individuals in Cluster 2 (Supplementary Figures S3F, G). Therefore, there is a high degree of coincidence among three computational methods of classification.

Univariate Cox analysis indicated that certain clinical variables, including age, M classification, N classification, T classification, TNM stage, and RMS exhibited an impact on the survival of BCa patients (Figure 6F). Above significant parameters were included into subsequent multivariate Cox regression analysis. The corresponding findings revealed that age >65 years (HR = 1.87, 95% CI: 1.33–2.64, *p* = 0.00032), advanced N classification (HR = 1.62, 95% CI: 1.17–2.25, *p* = 0.00403), and high RMS (HR = 1.61, 95% CI: 1.17–2.24, *p* = 0.00388) remained adverse and independent prognostic factor in BCa (Figure 6F). These findings imply that the RMS model is independent of conventional clinical variables and can predict the survival of BCa with comparatively satisfactory performance.

**FIGURE 7:**
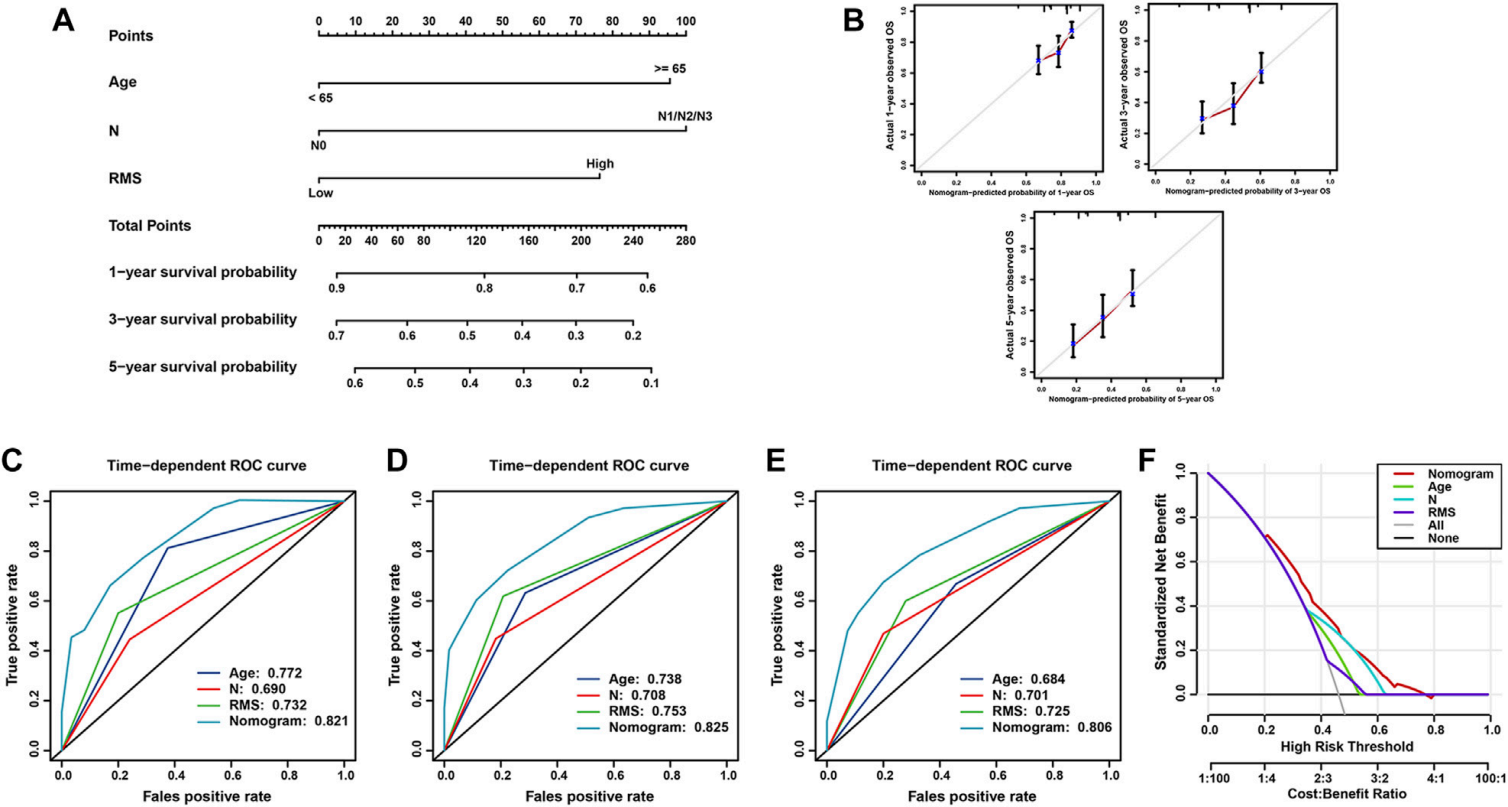
Construction and validation of nomogram model for survival prediction in BCa. **(A)** Nomogram for predicting the probability of 1-, 3-, and 5-year OS for BCa patients. **(B)** Calibration curve of nomogram to assess the consistency between predicted and observed 1-, 3-, and 5- year outcomes. Time-dependent ROC curve of the nomogram, N classification, age and the RMS for **(C)** 1-year, **(D)** 3-year, and **(E)** 5-year OS of BCa patients. **(F)** DCA curves of the nomogram model for BCa patients’ survival.

To confer physicians with a visualized approach to predict the long-term survival of BCa patients, the nomogram model encompassing the RMS signature and significant clinical risk factors identified by multivariate Cox analysis was formulated. As illustrated in Figure 7A, N classification made the greatest contributions to risk points, followed by age and the RMS model. The C-index of the nomogram was 0.785 (95% CI: 0.737–0.848) under 1,000 bootstrap replication. The calibration curves for the OS probability of 1-, 3-, and 5-year in BCa cases demonstrated a good agreement between nomogram prediction and practical observation (Figure 7B). The time-ROC curves were established to compare the predictive efficiency of this nomogram with that of N classification, age and the RMS. For the ROC curve of 1‐year survival, the AUC of nomogram (0.821) was higher than that of age (0.772), the RMS (0.732), and N classification (0.690) (Figure 7C). The nomogram to predict 3-year OS obtained the optimal AUC of 0.825, followed by the RMS (0.753), age (0.738), and N classification (0.708) (Figure 7D). The AUC for the nomogram, the RMS, N classification and age to predict 5-year survival were 0.806, 0.725, 0.701 and 0.684, respectively (Figure 7E). The decision curve analysis (DCA) of the nomogram was characterized with the optimal net benefits, followed by N classification, age, and the RMS (Figure 7F). In sum, the nomogram incorporating N classification, age and the RMS exhibits a relatively satisfactory predictive performance for the long-term survival of BCa patients.

### 3.7 Molecular subtypes and functional annotation associated with the RMS in BCa

**FIGURE 8:**
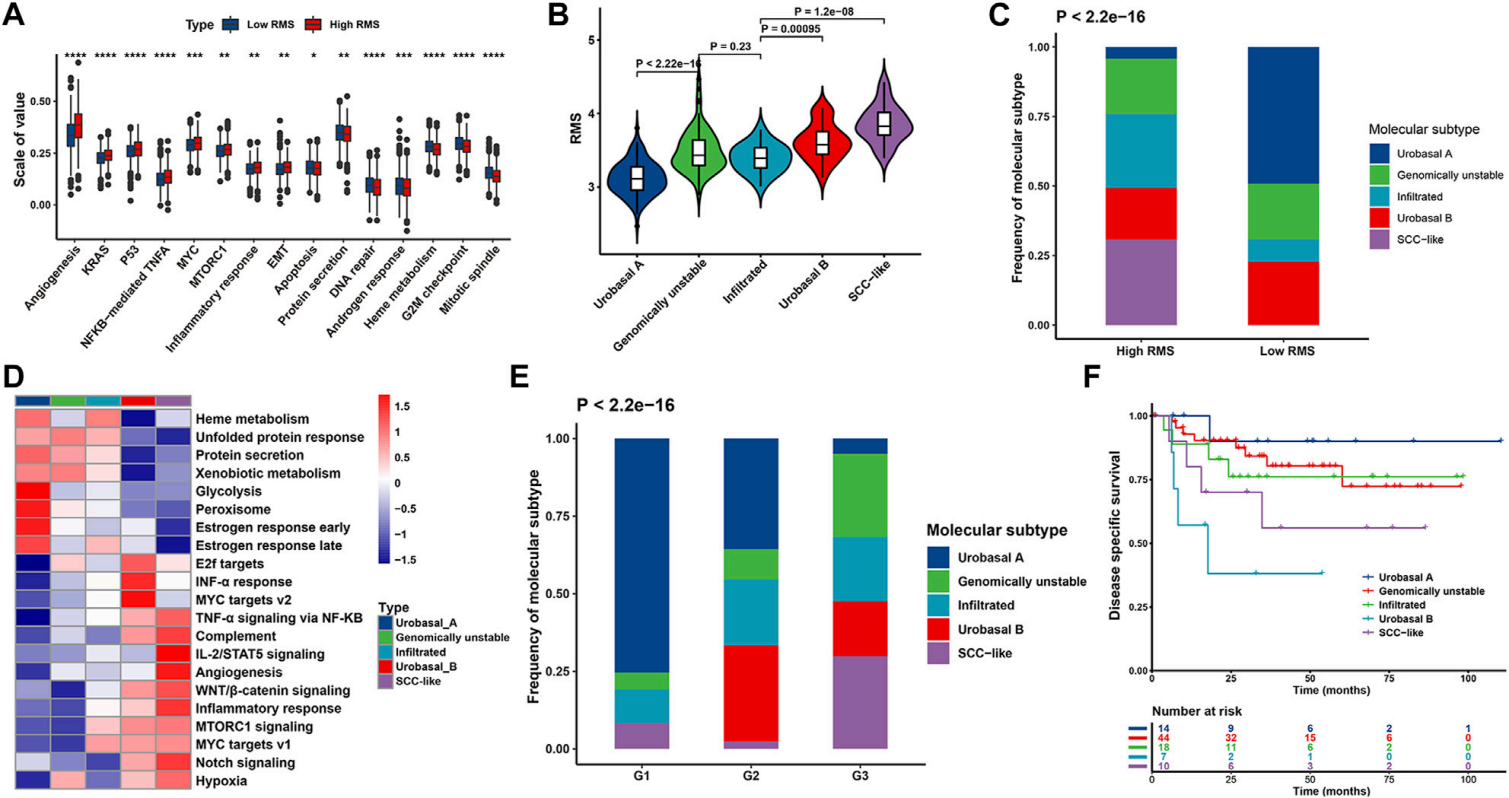
Biological function characteristics of the RMS model in BCa patients. **(A)** Boxplot representing the association between the RMS and known signatures based on the meta-GEO cohort. **(B)** Violin plot illustrating RMS distribution among five molecular subtypes based on GSE32894 **(C)** Bar chart describing difference in the distribution of five molecular subtypes between RMS-high and -low groups based on GSE32894 cohort. **(D)** Heatmap showing difference in the biological processes among five molecular subtypes. **(E)** Bar chart exhibiting difference in the distribution of histological grade among five molecular subtypes. **(F)** Kaplan-Meier curve revealing difference in OS among five molecular subtypes for BCa cases at histological grade 3.

We further illustrated the functional characteristics of the RMS signature through analyzing the association between the RMS model and known biological processes-associated gene sets identified by MSigDB (Subramanian et al., 2005), emphasizing that high RMS was significantly associated with stromal activation status and cancer progression-associated pathways, such as inflammatory response, NF-KB-mediated TNF-a, epithelial-mesenchymal transition (EMT), angiogenesis, IL-6/JAK/STAT3 signaling (Figure 8A and Supplementary Figure S3H).

Based on a comprehensive molecular subtypes landscape of UC established by Sjödahl et al.‘s study (Sjödahl et al., 2012), UC cases were classified into five molecular subtypes, including urobasal A (MS1 subdivided into MS1a and MS1b), genomically unstable (MS2a subdivided into MS2a1 and MS2a2), urobasal B (MS2b2.1), squamous cell carcinoma (SCC)-like (MS2b2.2), and one highly infiltrated by non-tumor cells (MS2b1). Notably, above molecular subtypes differed in survival patterns in which urobasal A exhibited favorable prognosis, genomically unstable and the infiltrated group were with moderate prognosis, and the urobasal B and the SCC-like were characterized with the shortest survival (Sjödahl et al., 2012). We compared the difference of the RMS among above five molecular subtypes through analyzing data downloaded from GSE32894. The SCC-like subtype showed the highest RMS, followed by the urobasal B, genomically unstable, the infiltrated subgroup and urobasal A (Figure 8B). Additionally, there was a significant discrepancy in the distribution of molecular subtypes between RMS-high and -low group. The urobasal A subtypes was primarily clustered in RMS-low group, conversely, the SCC-like and the infiltrated subtype were markedly concentrated on RMS-high group (Figure 8C).

To further decipher potential biological processes associated with different molecular subtypes of BCa patients in GSE32894, GSVA was performed implying that tumorigenesis-associated biological processes were significantly enriched in the SCC-like and the urobasal B subtype, including WNT, NOTCH, angiogenesis, and IL2-STAT5 signaling pathways. In contrast, the biological pathways activated in the urobasal A subtype were significantly correlated with the heme metabolism, protein secretion and peroxisome (Figure 8D). Consistently, the SCC-like and the urobasal B-related signaling pathways were prevailingly enriched in RMS-high cases, while the enrichment score of urobasal A-related biological processes were markedly clustered in RMS-low group (Supplementary Figure S3H). BCa patients in the urobasal B and the SCC-like subtype were prone to be diagnosed at more advanced stage compared with those in the urobasal A subtype (Figure 8E), which was also significantly correlated with diminished OS (HR = 12.3, 95% CI: 1.36–111, *p* = 0.026 for the urobasal B subtype) (Figure 8F). Previous results in our report demonstrated that the RMS positively correlated with BCa patients’ degree of malignancy. Thus, high RMS roughly corresponding to the urobasal B and the SCC-like subtype indicates unsatisfactory prognosis, which is potentially partly ascribed to the activation of EMT, WNT, angiogenesis, and additional signaling pathways mediating BCa tumorigenicity and tumor metastasis.

### 3.8 Pan-cancer analysis of RMS model-associated genes

Initially, we explored the correlation between CNV and mRNA expression in 14 RMS model-associated genes in 33 kinds of tumors and revealed that CHMP7 expression was significantly modulated by CNV in almost all cancers, followed by SEPHS1 and AASDHPPT (Supplementary Figure S4A). Specifically, a majority of RMS model-associated genes was characterized with heterozygous amplification of CNV in adenoid cystic carcinoma, while homozygous amplification was prone to occur in OV, esophageal cancer, and unconditioned stimulus (Supplementary Figure S4B). Thus, the findings highlight that the CNV of RMS model-associated genes is various among different tumors and it is essential to investigate the source of the heterogeneity. Furthermore, we explored the difference in mRNA expression between tumor and normal sample and revealed that the fold difference in the expression of RMS model-associated genes was the greatest in LUSC. Concretely, GDPD5 and IL28RA were significantly downregulated in BRCA than normal samples, while ROMO1 was overexpressed in BRCA samples (Supplementary Figure S4C). Additionally, pathway analysis demonstrated that RMS model-associated genes principally triggered cell cycle and EMT pathway while exerts an inhibitory effect on apoptosis and RAS/MAPK pathway (Supplementary Figure S4D). Therefore, our RMS model genes potentially plays a crucial role in malignant progression of tumors.

### 3.9 Difference in post-transcriptional events between RMS-high and -low groups in BCa

**FIGURE 9:**
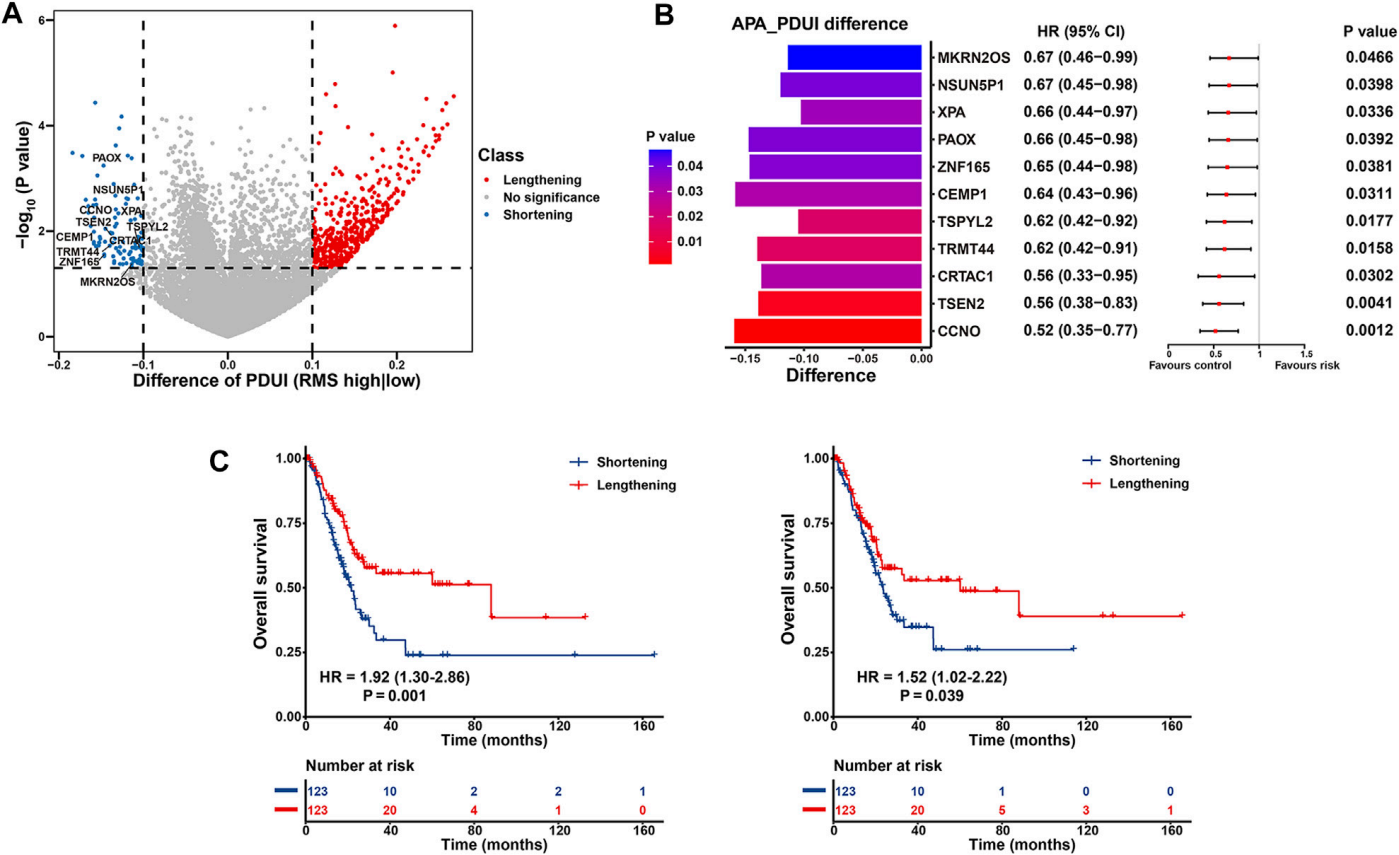
Post-transcriptional characteristics related to the RMS of BCa patients in the TCGA-BLCA cohort. **(A)** Volcano plot representing significantly differences in the PDUI of each gene between RMS-high and -low groups. **(B)** Bar graphs showing transcripts with shortening 3′UTR events in RMS-high group. Forest plots showing univariate Cox regression analysis for genes with differential PDUI between RMS-high and -low group. **(C)** Kaplan-Meier curve showing OS between PDUI lengthening and PDUI shortening of CCNO and PAOX.

To elucidate the functional effect of RNA modification “writers” on post-transcriptional characteristics of BCa patients, we investigated APA events of each gene in the TCGA-BLCA. Initially, we analyzed APA alterations between 246 BCa cases with high or low RMS and determined the prognostic significance of transcripts with significant 3′UTR alterations. A total of 11,598 APA events remained for differential analysis, and there were 503 genes with significantly lengthened 3′UTR (ΔPDUI >0.1) and 96 transcripts with markedly shortening 3′UTR (ΔPDUI <0.1) in RMS-high group, respectively (*p* < 0.05) (Figure 9A and Supplementary Table S12), and shortening APA events in RMS-high group were characteristic with worse OS based on univariate Cox regression analysis (Figure 9B and Supplementary Table S13), thus indicating that usage of a PAS may exacerbate BLCA malignancy. Specifically, the transcripts of *CCNO* (ΔPDUI = −0.16, *p* = 0.003) and *PAOX* (ΔPDUI = −0.15, *p* = 0.03) both underwent statistically significant 3′UTR shortening in patients with high RMS, which was associated with worse survival in BLCA (HR = 1.92, 95% CI: 1.30–2.86, *p* = 0.001 for *CCNO*; HR = 1.52, 95% CI: 1.02–2.22, *p* = 0.039 for *PAOX*) (Figure 9C). A report have demonstrated that *CCNO* is overexpressed in cervical squamous cell carcinoma (CSCC) and RACK1/miR-302b/c/d-3p-mediated CCNO inhibition can dampen the progression of CSCC (Wang and Chen, 2020). Suppression of *PAOX* is sufficient to widen the therapeutic index of cytotoxic drugs and overwhelm DNp73-mediated chemoresistance in cancers (Bunjobpol et al., 2014). Thus, we speculate that shortening 3′UTR of *CCNO* and *PAOX* in BLCA samples with high RMS potentially results in loss of several RNA regulatory elements, such as miRNA binding sites, thus enabling the upregulation of oncogenes expression and the progression of BLCA.

### 3.10 Potential role of the RMS in antitumor chemotherapy and antibody-drug conjugates (ADC) therapy

**FIGURE 10:**
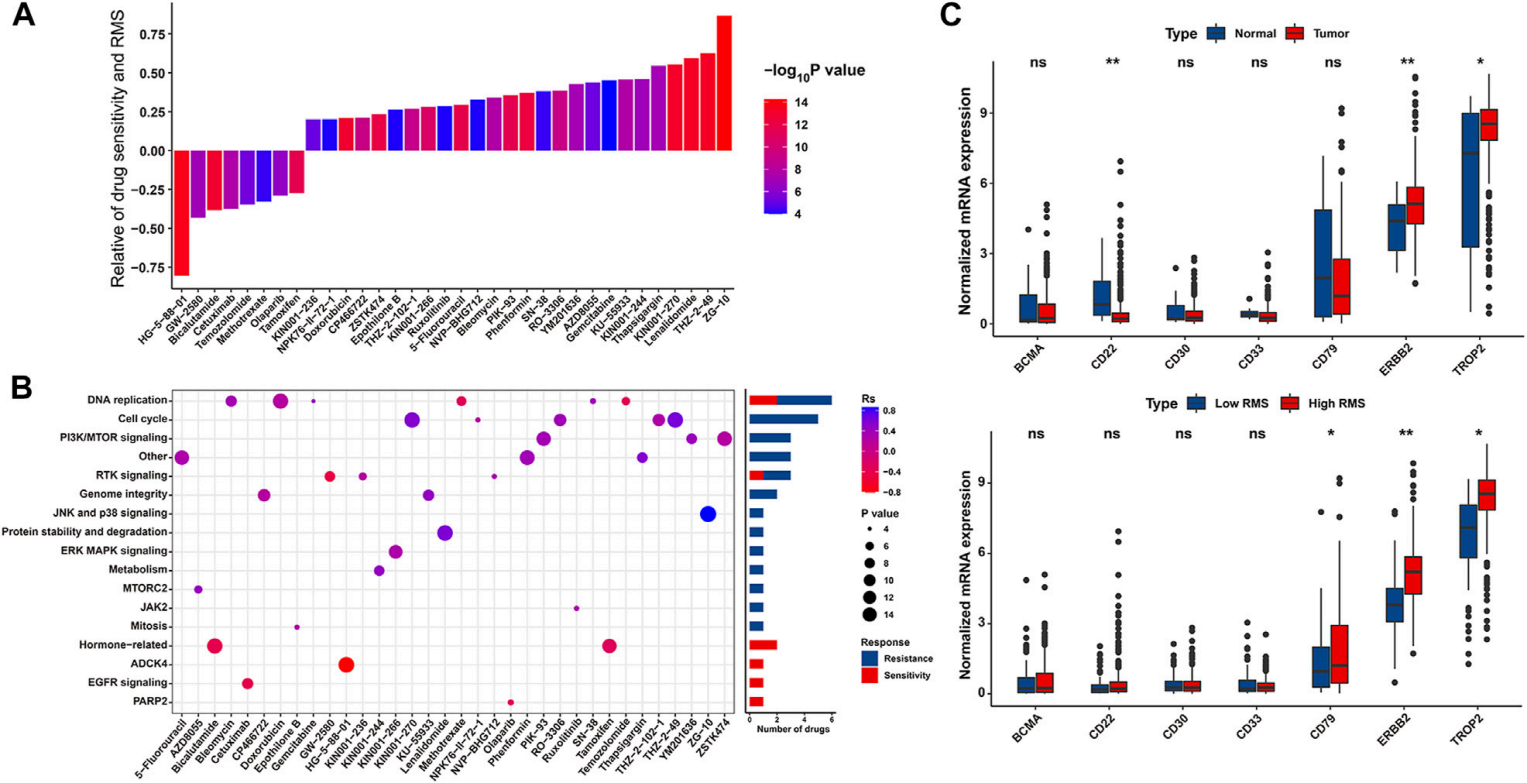
The association between the RMS and efficacy of antitumor chemotherapy. **(A)** Spearman correlation analysis between the RMS and drug sensitivity. The columns represented drugs. The brightness and height represented the significance and degree of the correlation, respectively. Rs > 0.2 or Rs < −0.2 indicated drug resistance or drug sensitivity, respectively. **(B)** Bar chart displaying signal pathways associated with drugs that were resistant (Blue) or sensitive (Red) to the RMS. *X* and *Y*-axis displayed drug names and corresponding signaling pathways, respectively. **(C)** Violin plot displaying mRNA levels of eight target antigens of ADC between normal tissues and BCa samples, and between RMS-low and -high BCa samples, respectively.

To further assess the relationship between the RMS and drug response of BCa cell lines, we determined 34 significantly correlated pairs between the RMS and drug response in the GDSC database based on Spearman correlation analysis (Yang et al., 2013). Specifically, there was significant correlation between drug sensitivity and the RMS in 8 pairs, including EGFR inhibitor HG-5–88–01 (Rs = −0.804, *p* = 0.005), CSF1R inhibitor GW-2580 (Rs = −0.43, *p* = 0.016), and AR inhibitor Bicalutamide (Rs = −0.383, *p* < 0.0001). Conversely, 26 pairs displayed drug resistance significantly related to the RMS, including JNK1 inhibitor ZG-10 (Rs = 0.867, *p* < 0.0001) and CDK9 inhibitor THZ-2–49 (Rs = 0.625, *p* < 0.0001) (Figure 10A and Supplementary Table S14). Additionally, we also explored the potential signaling pathways of drug-targeted genes. As revealed in Figure 10B, drugs whose sensitivity was linked to high RMS primarily targeted hormone-related, ADCK4, and EGFR signaling pathways, while drugs whose resistance was related to high RMS mostly targeted DNA replication, cell cycle and PI3K/MTOR signaling pathways. Thus, above findings indicate that RNA modification patterns are related to drug response of tumors. The RMS potentially develops into a novel biomarker to confer a reference for appropriate clinically interventional strategies.

Currently, certain ADCs have been approved by the US Food and Drug Administration (FDA) for the cancer therapy (Supplementary Table S15) (Bross et al., 2001; Krop et al., 2010; Senter and Sievers, 2012; Starodub et al., 2015; Trudel et al., 2019; Wynne et al., 2019; Modi et al., 2020; Sehn et al., 2020). Two target antigens (ERBB2 and TROP2) were lineage-specific markers of two out of above approved ADCs—Trastuzumab deruxtecan, and Sacituzumab govitecan, which have consistently high expression across the BCa tumor population than normal samples in the TCGA-BLCA (Figure 10C). We further evaluated the differences in the expression of seven target antigen molecules of ADC in RMS-low and -high groups. The target antigens, including ERBB2 and TROP2, were preferential expression on RMS-high BCa samples with a relative low expression on RMS-low subgroup (Figure 10C). Together, above findings implied that RNA modification patterns are potentially associated with ADC sensitivity.

### 3.11 Predictive value of the RMS in immunotherapeutic efficacy

**FIGURE 11:**
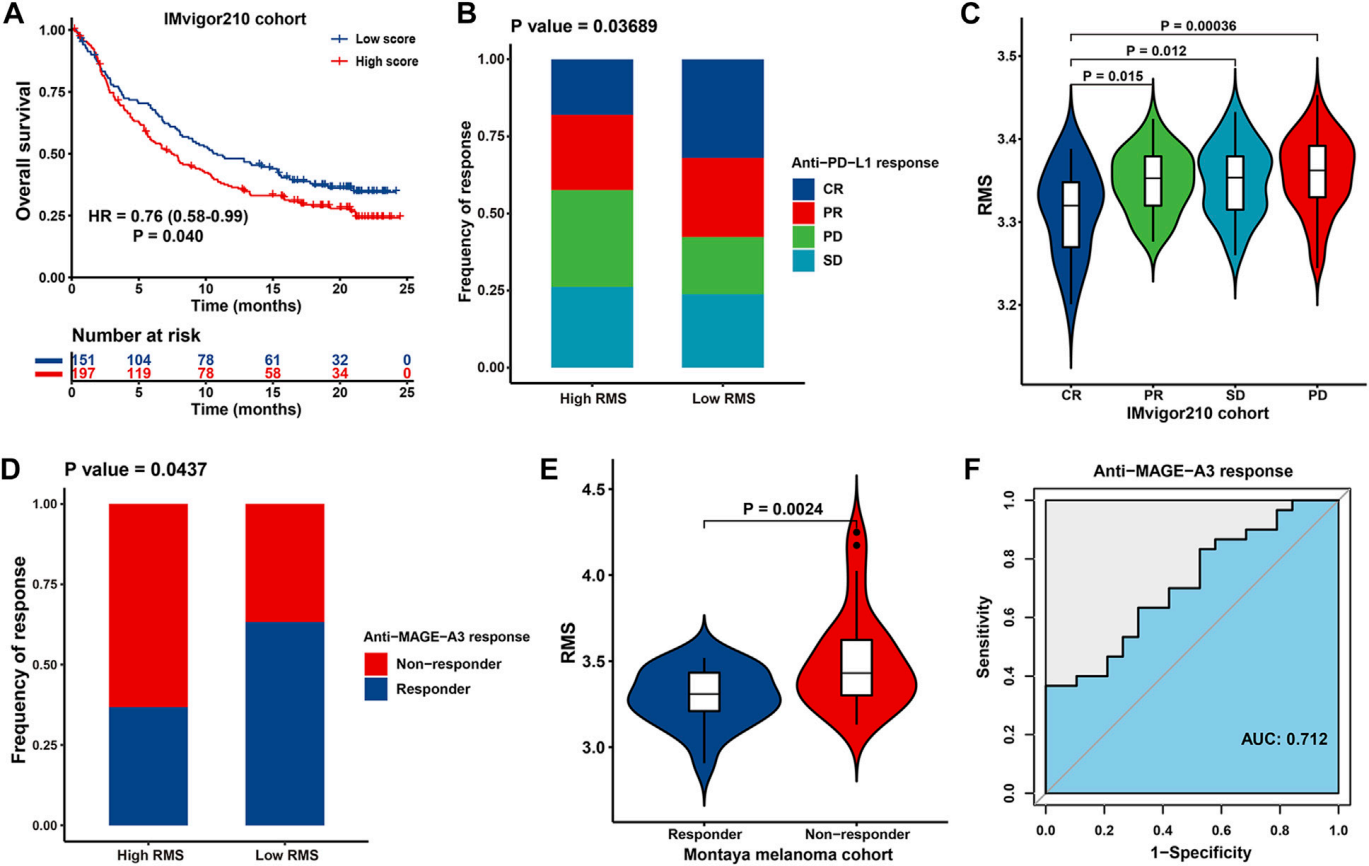
The association between the RMS and efficacy of immunotherapy in two cohorts. **(A)** Kaplan-Meier curve for OS of RMS-low and -high patients in IMvigor210 cohort. **(B)** Bar plot showing the fractions of different clinical responses to anti-PD-L1 immunotherapy in RMS-high or -low group in IMvigor210 cohort. **(C)** Violin plot displaying the distribution of the RMS in four groups about clinical response to anti-PD-L1 therapy in IMvigor210 cohort. **(D)** Bar plot revealing the proportions of different clinical responses to anti-MAGE-A3 immunotherapy in high/low RMS group in Montoya melanoma cohort. **(E)** Violin plot displaying the distribution of the RMS in four groups about clinical response to anti-MAGE-A3 treatment in Montoya melanoma cohort. **(F)** ROC curve describing the predictive performance of the RMS in evaluating patients’ response to anti-MAGE-A3 immunotherapy in Montoya melanoma cohort.

Immunotherapies of blocking T-cell inhibitory molecules PD-L1 and PD-1 have undoubtedly emerged a significant breakthrough in anticancer intervention. Meanwhile, it is urgent for us to make judgment about which subset of patients can benefit most from immunotherapies (Zeng et al., 2019). Therefore, we investigated the predictive power of the RMS for patients’ response to ICB therapy based on two immunotherapy cohorts. Patients with low RMS exhibited significantly prolonged OS than those with high RMS in IMvigor210 cohort (HR = 0.76, 95% CI: 0.58–0.99, *p* = 0.040) (Figure 11A). For IMvigor210 cohort, Chi-squared test demonstrated that compared with RMS-high group, RMS-low group was endowed with markedly increased proportion of the sum of CR and PR patients while significantly diminished the sum of PD and SD cases (*p* = 0.037) (Figure 11B). Likewise, CR patients were characterized with the lowest RMS compared with their counterparts with other types of response (Figure 11C). Significant therapeutic advantage and strengthened clinical response to anti-MAGE-A3 immunotherapy in patients with low RMS were also confirmed in Montoya melanoma cohort (Figures 11D, E). Additionally, we also validated the predictive performance of the RMS in anti-MAGE-A3 immunotherapy, with a satisfactory AUC value of 0.712 (Figure 11F). Collectively, cases with lower RMS are more possibly to reap better prognosis and enhanced clinical benefit from ICB therapy.

**FIGURE 12:**
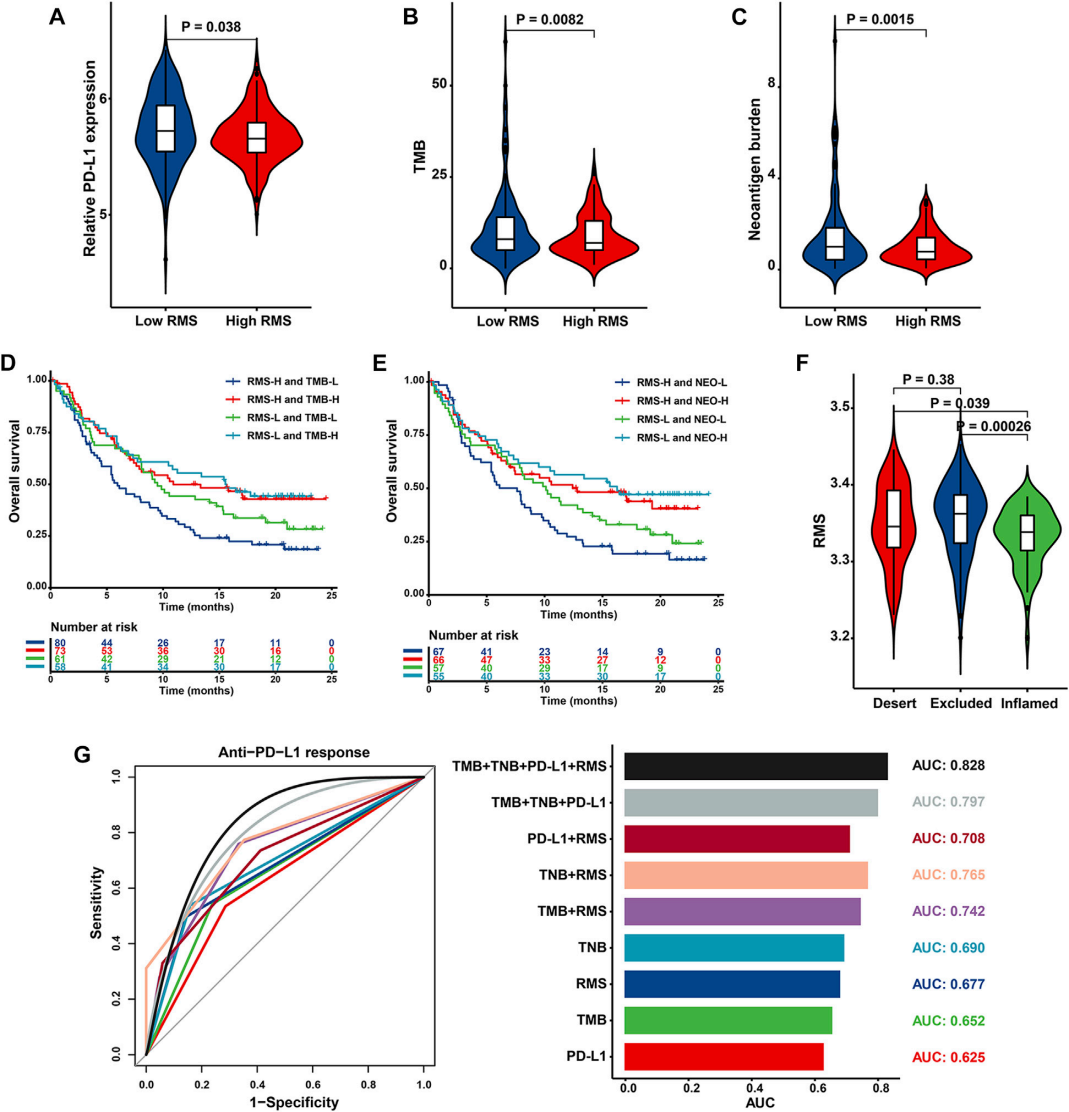
The biological significance and predictive value of RMS for the efficacy of anti-PD-L1 immunotherapy in IMvigor210 cohort. Violin plot depicting differences in the levels of **(A)** PD-L1, **(B)** TMB, and **(C)** neoantigen burden between RMS-low and -high group. Kaplan-Meier curve showing OS of multiple subgroups stratified by the RMS and **(D)** TMB or **(E)** neoantigen burden **(F)** Violin plot presenting difference in the RMS among three immune phenotypes. **(G)** Histogram and ROC curve displaying the predictive power of nine signatures composed of TMB, TNB, RMS and PD-L1.

Accumulated evidence has emphasized that patients with elevated TMB, higher neoantigen burden, certain DNA repair mutations, mismatch repair deficiency, and higher PD‐L1 expression level are correlated with improved objective response, durable clinical benefit, and prolonged long-term survival when receiving ICB therapy (Rizvi et al., 2015; Le et al., 2017). Based on tumor-associated immune phenotypes depicted in IMvigor210 cohort, patients with low RMS were characterized with significantly increased PD-L1 level (Figure 12A). Similarly, cases in RMS-low group had significantly strengthened TMB and neoantigen burden than those with high RMS (Figures 12B, C), indicating a potential response to ICB. Patients with the combination of low RMS and high TMB/neoantigen burden displayed the optimal survival advantage (HR = 0.51, 95% CI: 0.33–0.79, *p* = 0.003 for Low RMS with high TMB; HR = 0.48, 95% CI: 0.31–0.76, *p* = 0.002 for Low RMS with high neoantigen burden) (Figures 12D, E). We further explored the difference in the RMS among three phenotypes, including “immune inflamed”, “immune excluded”, and “immune desert” (Chen and Mellman, 2017). As illustrated in Figure 12F, patients with an immune-inflamed phenotype exhibited the lowest RMS compared with the other two phenotypes. Above findings partly explain why immunotherapy is prone to exert intensive antitumor effect in the low RMS subset. Our aforementioned results also demonstrated that MDSC which is recognized to mediate immune tolerance in the TME was significantly activated in RMS-high group, indicating that RMS-high tumors potentially represent “cold tumors” with resistance to immunotherapy. Furthermore, AUC value evaluating the capacity of the RMS model, TMB, TNB and PD-L1 to differentiate responders from non-responders was 0.677 (95% CI = 0.589–0.765), 0.652 (95% CI = 0.549–0.755), 0.690 (95% CI = 0.595–0.785), and 0.625 (95% CI = 0.517–0.733), respectively. The results also illustrated that the RMS in combination with TMB, TNB and PD-L1 had the optimal predictive power, with the highest AUC of 0.828 (95% CI = 0.714–0.941), followed by TMB combined with TNB and PD-L1 (AUC = 0.797, 95% CI = 0.678–0.916), the RMS combined with TNB (AUC = 0.765, 95% CI = 0.671–0.859), the RMS combined with TMB (AUC = 0.742, 95% CI = 0.641–0.843), and the RMS combined with PD-L1 (AUC = 0.708, 95% CI = 0.595–0.822) (Figure 12G). Briefly, these results may introduce the novel piece to the atlas of RNA modification patterns’ influence on the efficacy of immunotherapy.

## 4 Discussion

The building blocks of RNA are canonically confined to four bases, nevertheless, RNA modifications can tremendously expand the chemical diversity of RNA. It is therefore not surprising that RNA modifications have attracted much attention recently owing to their sophisticated and widespread impacts on inflammation, innate immunity, antitumor activity, and the response to immunotherapy through the cross-talk among multifarious “writers”. With the exception of certain studies centralized a single type of RNA modification “writer”, there has been no literature so far of a comprehensive elucidation in the multifaceted association and effects of diverse types of “writers” on malignancy (Lan et al., 2019; Xing et al., 2021). Therefore, in our report, we unveiled the global profiling of RNA modification patterns and its impact on prognostic characteristics and immune landscape in the TME of BCa and further combined the RNA modifications-related model and additional routine clinicopathological indicators to effectively predict the clinical outcome and immunotherapy effectiveness, which potentially opens up a new dimension for the management of BCa.

The “writers” of RNA modification exerts momentous impacts on normal growth and their mutation or disharmony is related to both genetic disorders and multiple malignancies (Zhang et al., 2015). Herein, we described the mutation landscape of 34 “writers” and its prognostic role in BCa for the first time. We found that m^6^A “writers” METTL3 and KIAA1429 and APA enzymes PCF11 were more predisposed to mutation than additional “writers” in BCa, while mutations of the “writers” CPSF1, ADARB2 and KIAA1429 were proved to be more frequent in hepatocellular carcinoma (HCC) and the mutation frequency of the “writers” ZC3H13, PCF11 and KIAA1429 was the highest in colorectal cancer (CRC) (Qian et al., 2019; Xing et al., 2021). We also observed that mutations of 34 “writers” genes were correlated with worse OS of BCa patients, making it clear that total diminished level of RNA modification is endowed with a crucial role in BCa development. Similarly, a shorter OS in clear cell renal carcinoma (ccRCC) patients with “writers” genes loss of function was revealed (Qian et al., 2019). The carcinogenic activation pathways were significantly enriched in HCC cases with the “writers” mutation, indicating the relationship between the mutant status of “writers” and worse outcomes of HCC patients (Xing et al., 2021). Intriguingly, CRC cases with mutant “writers” had poorer prognosis compared with those without mutations (Chen et al., 2021). Thus, the discrepancies of mutant status of “writers” and its associated prognostic effects between different tumor types gave us a clue that the modulation of RNA modification in cellular level was sophisticated, and more researches concentrating on the “writers” are required to further illustrate the regulatory mechanism of RNA modification in BCa.

We specifically summarized global alterations of m^6^A, m^6^A_m_, m^1^A, APA, and A-to-I RNA editing enzymes at transcriptional and genetic levels and their mutual correlation in BCa. Specifically, m^6^A “writer” KIAA1429 was the third most common mutant gene and had relatively prevalent CNV gains, with a negative association with the prognosis of BCa patients, indicating the potential function of KIAA1429 in promoting carcinogenesis and metastasis. Prior studies have confirmed significant overexpression of KIAA1429 in multifarious human cancers, including hepatocellular carcinoma (HCC) (Lan et al., 2019), breast cancer (Qian et al., 2019), non-small cell lung cancer (NSCLC) (Tang et al., 2021), gastric cancer (Miao et al., 2020), and osteosarcoma (Han et al., 2020), which was positively correlated with malignant biological properties while linked to significantly diminished OS of above tumors. Mechanistically, KIAA1429-mediated m6A methylation on the 3′UTR of GATA3 pre-mRNA elicits the separation of HuR and the resulting degradation and downregulation of GATA3, which triggers HCC development (Lan et al., 2019). Furthermore, KIAA1429 is sufficient to enhance the expression of CDK1 by an m^6^A-independent manner and further accelerates breast cancer progression (Qian et al., 2019). KIAA1429 favors the mRNA stability of HOXA1 *via* targeting its 3′UTR to confer NSCLC on gefitinib resistance, suggesting the role of KIAA1429 as potential therapeutic target in NSCLC (Tang et al., 2021).

In addition to elucidating the specific role of individual RNA modification “writer” in the prognosis and immunity of BCa, we also investigated the clustering result of 34 RNA modification “writers”. Two distinct RNA modification patterns (Cluster 1 and Cluster 2) were identified based on 34 RNA modification enzymes. We confirmed that MDSCs and Th17 cells were accumulated in Cluster 1 cases that was characterized with poor survival and low response rate to immunotherapy. MDSCs are a cluster of pivotal immunosuppressive cells in the TME, which are endowed with the capacity to impede T cell, NK cell and B cell functions partly through stimulating the expression of ARG1, indoleamine 2, 3-dioxygenase and inducible nitric oxide synthase (Veglia et al., 2018). MDSCs also interact with tumor cell and foster its stemness characteristics, thereby maintaining a malignant phenotype of tumors (Schneider et al., 2019). MDSCs secrete diversiform chemokine receptors that are implicated in their recruitment to the TME, such as CXCR4 or CXCR2, as revealed in BCa patients (Obermajer et al., 2011; Zhang et al., 2017). Therefore, blocking the recruitment of MDSCs to the TME or depleting MDSCs in the tumor is a potentially promising strategy. Previously reports have demonstrated that patients with tumor who have high levels of circulating MDSCs exhibit an undesirable response rate to immunotherapy (Schneider et al., 2019). Intriguingly, a chemical agonist LXR-mediated the activation of ApoE secretion devastates MDSC survival by facilitating the binding of ApoE to its receptor LRP8, resulting in a fortified anti-tumor response (Tavazoie et al., 2018). A phase I clinical trial in BCa patients, is currently testing a LXR agonist (RGX-104) as a single agent or combined with nivolumab to strengthen the anti-tumor activity and the response to anti-PD-1 therapy (Schneider et al., 2019). Pathologically, Th17 response participates in certain inflammatory events, autoimmune and allergic diseases. Th17 cells have been demonstrated in increased levels in certain tumors, it remains controversial whether IL-17 facilitates or suppresses tumor progression. Specifically, IL-17-induced the generation of IL-6 activates oncogenic STAT3 and subsequently accelerates the expression of pro-survival and angiogenic genes, resulting in the development of BCa (Wang et al., 2009).

We then formulated a scoring model, RMS, to evaluate the efficacy of RNA modification “writers” in each case. RMS-high group was related to worse prognosis, which was partly attributed to significant activation of EMT, Notch, IL-2/STAT5, IL-6/JAK/STAT3, angiogenesis signaling pathways, which was instrumental in tumor invasion. The biological process of EMT involves epithelial cells assuming a mesenchymal phenotype, with reinforced capability for invasion and metastasis to accelerate malignant progression of BCa (Wang et al., 2018). In studies encompassing a wide spectrum of malignancies, including prostate cancer, breast cancer, and multiple myeloma, there is adamant evidence holding a crucial effect of Jagged-mediated Notch signaling on tumor metastasis. Notch activation drives FOXC2-dependent metastasis in PTEN-null prostate cancer mice (Kwon et al., 2016; Majumder et al., 2021). A paracrine loop composed of TGF-β and Jagged-mediated Notch activation also facilitates osteolytic bone metastasis in breast cancer (Sethi et al., 2011). Activation of IL-2/STAT5 signaling converged on an enhancer (CNS0) potentiates the generation and accumulation of IL-2 dependent thymic Treg cell lineage, potentially dampening host immune responsiveness (Dikiy et al., 2021). In the pathogenesis of cancer, increased IL-6 directly on stimulate cells in the TME to upregulate STAT3 target genes, subsequently driving the expression of proliferation-promoting proteins (such as cyclin D1), survival-associated molecules (such as BCL2-like protein 1), angiogenic factors (such as VEGF), invasiveness and metastasis-related proteins (such as matrix metalloproteinases) and immunosuppressive molecules (such as IL-10 and TGF-β) (Johnson et al., 2018).

ADCs are novel targeted agents that concatenate a cytotoxic drug (also known as cytotoxic payload or warhead) by a linker to a monoclonal antibody (mAb) which can specifically reach target antigens expressed on cancer cellular surface and deliver a potent cytotoxic payload to the tumor location, ultimately strengthening the chemotherapeutic efficacy and minimizing toxicity to normal tissue. The target antigen should be abundantly expressed on tumor cells while is not expressed or at a low level in normal tissues in an ideal setting, thus lowering off-target toxicity (Hafeez et al., 2020). Recent clinical progressions in the antibody-drug conjugates field provide promising potentialities for the future utility of the ADC agents as targeted treatment for patients with various malignancies. By 2022, enfortumab vedotin (EV) and sacituzumab govitecan (SG) are the only ADCs to obtain approval for the therapy of mUC. EV consists of a monoclonal antibody (mAb) specifically targeted transmembrane protein nectin-4 which is generally overexpressed in mUC and exerts a pivotal effect on cell-cell adhesion (Challita-Eid et al., 2016). SG is an ADC composed of a mAb specific for TROP2 conjugated *via* the topoisomerase inhibitor SN-38. Trop-2, a transmembrane calcium signal transducing glycoprotein, plays an integral part in cell growth and migration and is upregulated in various epithelial tumors including UC (Tagawa et al., 2021). In our report, we investigated whether the RMS could predict the efficacy of the ADCs for the first time. Collectively, our report deepens the comprehension of the modulation of RNA modifications in the TME of BCa and is conducive to the development of novel predictive indicators for patient stratification, prognosis evaluation, and personalized therapy in BCa.

Eventually, considering the remarkable effect of RNA modification patterns on immune infiltration in the TME, we showed enormous interest in the capacity of the RMS to predict the potential therapeutic effects of ICB therapy. Our findings highlight that the RMS was a potent predictor to assess the clinical outcome of distinct immunotherapy regimens (anti-PD1/L1 or anti-MAGE-3), which was validated in two UC immunotherapy cohort and two melanoma immunotherapy cohorts. The RMS combined with TMB could differentiate non-responders who underwent immunotherapy from responders with a more robust capability and a remarkably increased accuracy. Thus, our results allow the development of personalized cancer immunotherapy and advance our capacity to exploit an additional approach through which the response rate of immunotherapy can be enhanced.

Specific innovativeness and advantages should be emphasized in our report. Firstly, thus far, an impressive number of studies primarily focus on the importance of only one type of RNA modification (especially m^6^A) in biological processes and tumor pathogenesis. Considering that epitranscriptome embraces various RNA modifications and a direct interaction exists between the most abundant RNA modifications such as m6A and A-to-I (Xiang et al., 2018a; Tassinari et al., 2021), we elucidated the potential link between five forms of RNA adenosine modifications (including m^6^A, m^6^Am, m^1^A, APA, and A-to-I editing) and the prognostic characterization and immunologic landscape in BCa for the first time. Additionally, numerous studies have documented the modulators in RNA modification pathways, including “writers”, “erasers” and “readers”. Among these modulators, “writers” exert a major catalytic role and install the methylation in RNA modification process. We comprehensively summarized and identified 34 RNA modification “writers” from all relevant published literature. The consensus clustering results for 34 “writers” are satisfactory, thanks to the potential synergistic effect of “writers”. Herein, for the first time, we demonstrated that the mutant landscape, expression level, immune modulation, prognostic significance, and tumor-related pathways of single “writer” (such as KIAA1429) in BCa, which sets the stage and heightens interest in comprehending the biological function and underlying mechanisms of RNA modifications “writer” in BCa. Secondly, a total of 1801 BCa patients were incorporated into our report. Eight independent GEO datasets consisting of 1,410 BCa cases were merged into one meta‐GEO as training cohort. The TCGA-BLCA dataset was considered as independent validation cohort to externally validate the robustness and application of our RMS model. Thus, our model was developed and validated in varying platforms and large populations, which can be served as a promising prognostic signature to optimize BCa patient management. Also, WGCNA-based analysis determined the most weighted prognostic marker (KIAA1429) and its expression levels in BCa were analyzed *via* human tissue samples, thus guaranteeing the dependability of the results in this report. Thirdly, based on multiple ICB therapy cohorts, we validated that RNA modifications-related model could efficiently predict the efficacy of immunotherapy and might achieve optimal predictive performance when combined with traditional indicators (including TMB, TNB, and PD-L1).

Despite its promising results, several limitations should be mentioned in our study. Firstly, we merely utilized a median cutoff of the RMS based on the meta-GEO cohort to stratify BCa patients. The results need to be validated in a prospective cohort of patients treated with immunotherapy, thus more comprehensively defining the cutoff value to be used. Furthermore, considering the primary clinical significance of distinct tumor regions, it is necessary to systematically assess immune characteristics in the core of the tumor and at the invasive margin. Because not all cases with low RMS exhibit enduring and effective response to immunotherapy, other clinicopathological parameters should be included into the model to improve predictive performance. Thirdly, the special role and underlying mechanism of novel predictive indicators in the RMS model require further experiment research.

## 5 Conclusion

In summary, our profound and comprehensive analysis of five forms of RNA modification “writers” highlighted an extensive modulatory mechanism by which they exert effects on TME and their correlation with BCa prognosis. We determined two distinct RNA modification-associated subtypes in BCa and constructed an individual RNA modification “writers” profile scoring system that unraveled the interplay and regulatory roles of the “writers” in BCa prognosis, molecular subtypes and post-transcriptional events and depicted their predictive performance in chemotherapy, ADC therapy and immunotherapy. Our study emphasizes the pivotal clinical significance of the interaction among RNA modifications and advances our capacity to guide more effective and personalized immunotherapy for BCa.

## Data Availability

The original contributions presented in the study are included in the article/Supplementary Material, further inquiries can be directed to the corresponding author.

## Supplementary material

Supplementary Figure S1 Biological and expressional characteristics of RNA modification “writers” in BCa. **(A)** Violin plot displaying expression levels of “writers” in different subgroups stratified by CNV values in the TCGA-BLCA cohort. **(B)** Forest plot representing OS with HR of 34 RNA modification “writers” using univariate Cox regression analysis in the TCGA-BLCA cohort.

Supplementary Figure S2 Consensus clustering of BCa patients in the meta-GEO cohort. **(A)** Consensus clustering matrix of 1410 BCa cases for k = 2, 3, 4, 5, and k = 2 displayed the optimal RNA modification patterns. **(B)** Boxplot showing the relative expression of RNA modification “writers” in two patterns. **(C)** Boxplot illustrating difference in the abundance of infiltrating immune cells between Cluster 1 and Cluster 2.

Supplementary Figure S3 Enrichment analysis of DEGs and the association between prognosis and the RMS of BCa patients. **(A)** Bar chart depicting significant biological processes associated with RNA modification phenotype-related DEGs by GSVA. **(B)** Tenfold cross-validation to choose the tuning parameters in the LASSO regression. Two perpendicular dashed lines were drawn at the optimal values based on the minimum criterion (Right) and the 1-SE criterion (Left). **(C)** LASSO coefficient profiles of the 14 candidate DEGs used to establish the RMS. **(D)** Kaplan-Meier curve of DSS in patients with high or low RMS in GSE32894. **(E)** Kaplan-Meier curve of RFS in patients with high or low RMS in GSE31684. **(F)** Venn diagram and **(G)** Bar chart showing the intersection between the RMS-high/-low and Cluster 1/2. **(H)** Heatmap showing difference in the biological processes between RMS-high and -low group in GSE32894 cohort.

Supplementary Figure S4 Pan-cancer analysis of RMS model-associated genes. **(A)** Dot chart showing RMS model-associated genes whose mRNA expression was significantly correlated with CNV percentage. Blue and red bubbles represented a negative or positive correlation between mRNA expression and CNV. The color and size bubble illustrated the degree of correlation and the statistical significance, respectively. **(B)** Pie diagram illustrating proportion of different types of CNV of RMS model-associated genes in multiple tumors. **(C)** Dot chart representing mRNA differential expression analysis of 14 RMS model-associated genes between multiple tumors and normal samples. The colors from purple to red represented the fold change between tumor and normal tissues. **(D)** Heatmap revealing RMS model-associated genes that have inhibitory or activated functions in multiple cancers. Pathway_A (Red) and pathway_I (Blue) represented the proportion of tumor in which pathways may be activated or inhibited by model-related genes, respectively. Abbreviation: Hete Amp, heterozygous amplification; Hete Del, heterozygous deletion; Homo Amp, homozygous amplification; Homo Del, homozygous deletion.

## Glossary

95% CI: 95% confidence interval
3′UTR: 3′-untranslated region
**m** ^**6**^ **A**: N^6^-methyladenosine
**m** ^**6**^ **A**_**m**_: N^6^,2′-O-dimethyladenosine
**m** ^**1**^ **A**: N^1^-methyladenosine
APA: alternative polyadenylation
ADC: antibody-drug conjugates
A-to-I: adenosine-to-inosine
Am: 2′-O-methyladenosine
AUC: an area under the curve
ADAR: adenosine deaminase acting on RNA
BCa: bladder cancer
CSCC: cervical squamous cell carcinoma
CR: complete response
ceRNA: competing endogenous RNA
C-index: concordance index
CNA: somatic copy number variation
DSS: disease-specific survival
DEMs: differentially expressed miRNAs
DEGs: differentially expressed genes
DC: dendritic cell
DDR: DNA damage repair
EMT: epithelial-mesenchymal transition
FC: fold change
FDR: false discovery rate
GO: Gene Ontology
GSEA: gene set enrichment analysis
GSVA: Gene set variation analysis
GEO: Gene Expression Omnibus
HR: hazard ratio
IHC: Immunohistochemistry
ICB: Immune-checkpoint blockade
KEGG: Kyoto Encyclopedia of Genes and Genomes
LASSO: least absolute shrinkage and selection operator
miRNA: microRNA
MDSC: Myeloid derived suppressor cell
MSigDB: Molecular Signature Database
miRNA-seq: miRNA-sequencing
m7G: N7-methylguanosine
NES: normalized enrichment score
NK cells: natural killer cells
OS: overall survival
PAS: proximal polyA sites
PD-1: programmed cell death-1
PDUI: Percentage of Distal polyA site Usage Index
PCA: principal component analysis
Pan-FTBRS: pan-fibroblast TGF-β response signature
PD: progressive disease
RNA-seq: RNA-sequencing
PR: partial response
RFS: recurrence-free survival
ROC: receiver operating characteristic
RMS: RNA modification “writers” score
SD: standard deviation
snRNAs: small nuclear RNAs
SCC: squamous cell carcinoma
SD: stable disease
TMM: Trimmed Mean of M
TMA: tissue microarray
TISIDB: Tumor and Immune System Interaction Database
TME: tumor microenvironment
TMB: tumor mutation burden
Th17 cell: Type 17 T helper cell
TCGA-BLCA: The Cancer Genome Atlas-Bladder Urothelial Carcinoma
UC: urothelial carcinoma
UCSC: University of California Santa Cruz
WGCNA: Weighted gene co-expression network analysis
**“Writers”** ^**MUT**^: “writers” mutated
**“Writers”** ^**WT**^: “writers” wild-type
