# Supplementary figures and images for: Comprehensive analysis of a novel RNA modifications-related model in the prognostic characterization, immune landscape and drug therapy of bladder cancer
Source: Front Genet. 2023 Apr 12;14:1156095. doi: 10.3389/fgene.2023.1156095 (PMC10131083; doi:10.3389/fgene.2023.1156095)

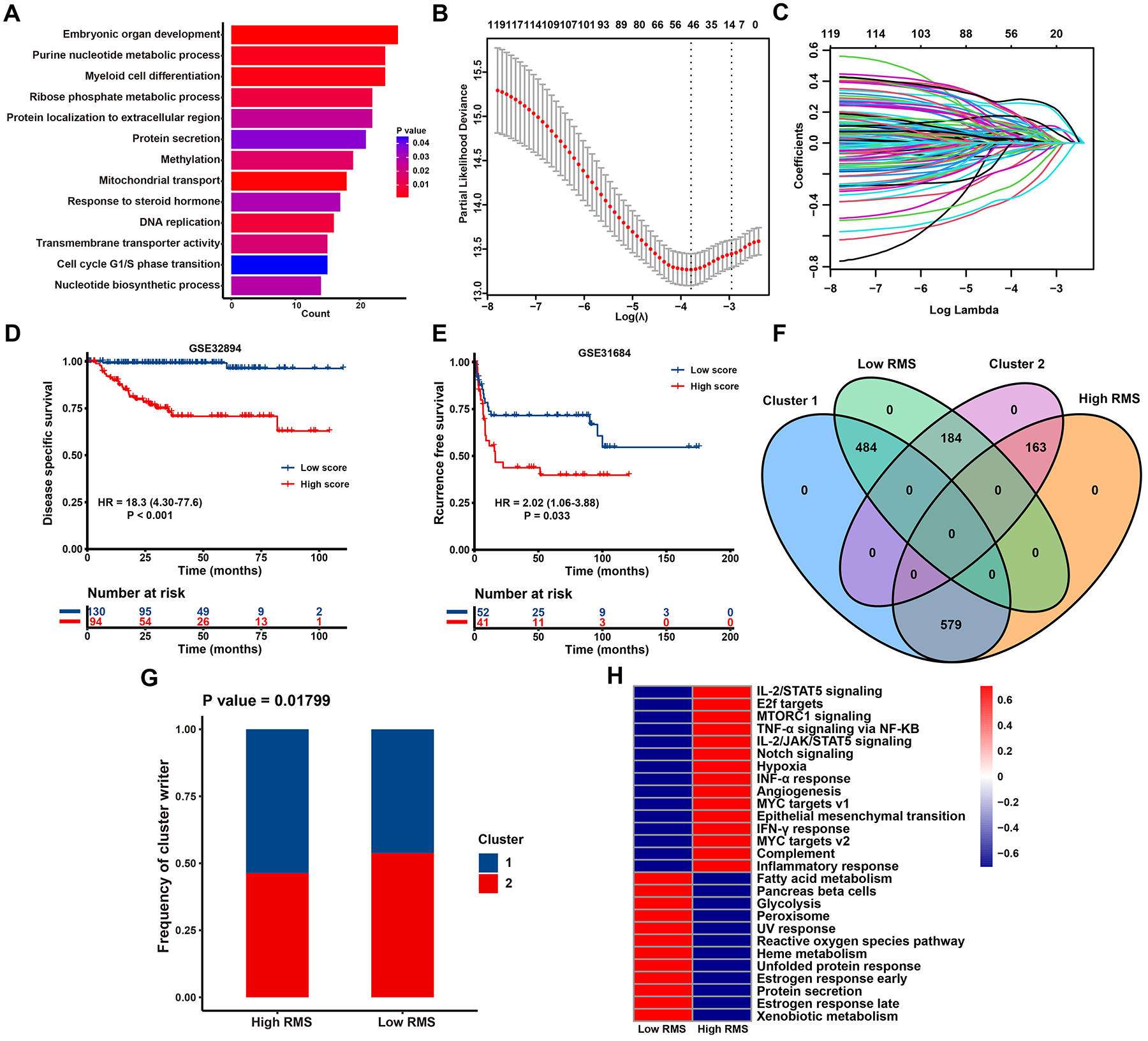

Supplement: Supplementary file 1 [file Image3.TIF]

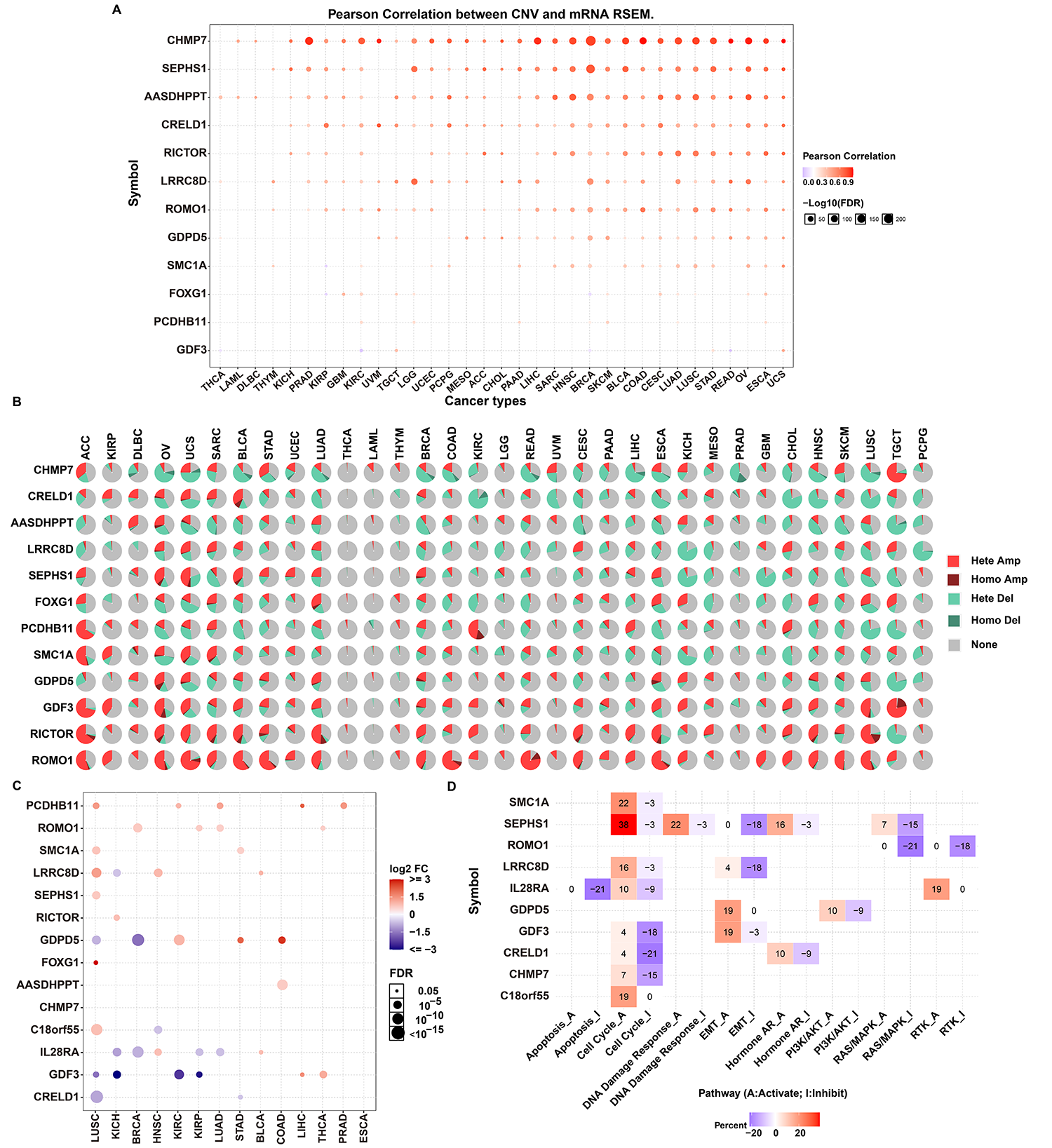

Supplement: Supplementary file 2 [file Image4.TIF]

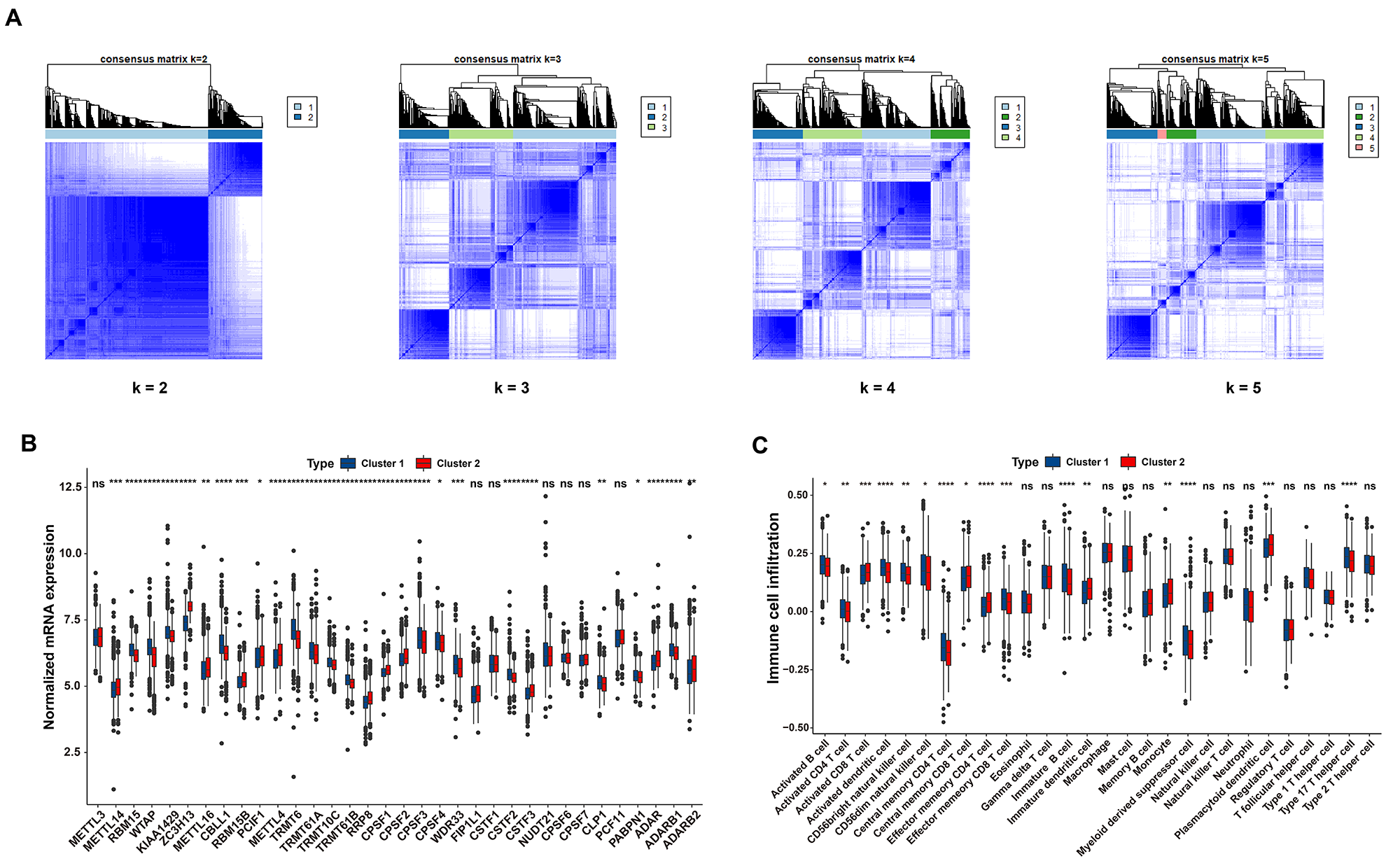

Supplement: Supplementary file 3 [file Image2.TIF]

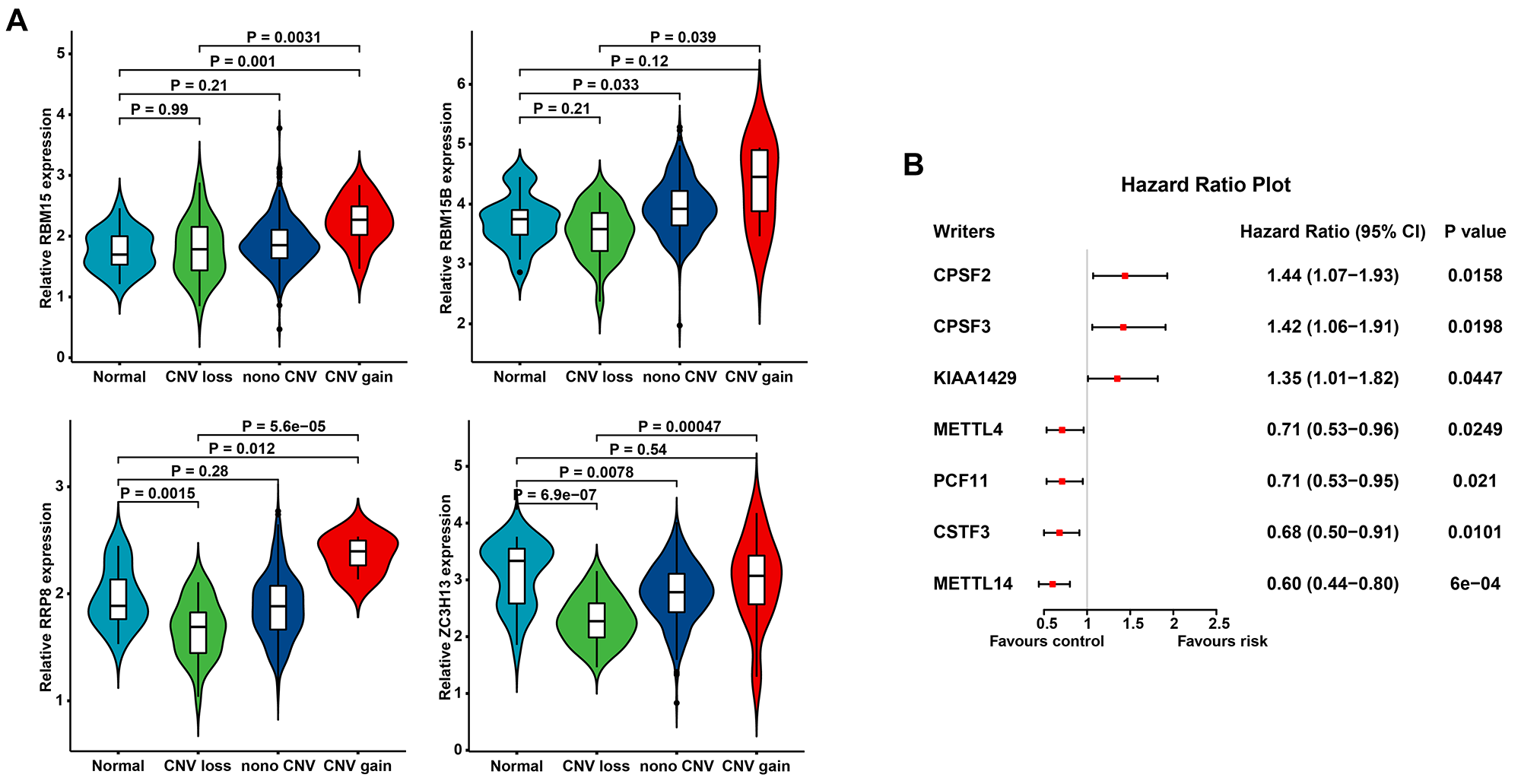

Supplement: Supplementary file 4 [file Image1.TIF]
